# Design of Anticancer 2,4-Diaminopyrimidines as Novel Anoctamin 1 (ANO1) Ion Channel Blockers

**DOI:** 10.3390/molecules25215180

**Published:** 2020-11-06

**Authors:** Taewoo Kim, Sinyoung Cho, Haejun Oh, Joonseong Hur, Haedong Kim, Young-Ho Choi, Seongho Jeon, Young Duk Yang, Seok-Ho Kim

**Affiliations:** 1 Department of Pharmacy, College of Pharmacy and Institute of Pharmaceutical Sciences, CHA University, 120 Haeryong-ro, Pocheon 11160, Korea; taewookim@snu.ac.kr (T.K.); jsy7122@naver.com (S.C.); dhgowns2@naver.com (H.O.); eanby12@nate.com (H.K.); dudgh705@gmail.com (Y.-H.C.); cmb_jsh@naver.com (S.J.); 2 Natural Products Research Institute, Korea Institute of Science and Technology (KIST), 679 Saimdang-ro, Gangneung 25451, Korea; hjs1120@kist.re.kr

**Keywords:** anoctamin 1, ion channel blocker, pyrimidine, library, anti-proliferative

## Abstract

Pyrimidine is a privileged scaffold in many synthetic compounds exhibiting diverse pharmacological activities, and is used for therapeutic applications in a broad spectrum of human diseases. In this study, we prepared a small set of pyrimidine libraries based on the structure of two hit compounds that were identified through the screening of an in-house library in order to identify an inhibitor of anoctamin 1 (ANO1). ANO1 is amplified in various types of human malignant tumors, such as head and neck, parathyroid, and gastrointestinal stromal tumors, as well as in breast, lung, and prostate cancers. After initial screening and further structure optimization, we identified **Aa3** as a dose-dependent ANO1 blocker. This compound exhibited more potent anti-cancer activity in the NCI-H460 cell line, expressing high levels of ANO1 compared with that in A549 cells that express low levels of ANO1. Our results open a new direction for the development of small-molecule ANO1 blockers composed of a pyrimidine scaffold and a nitrogen-containing heterocyclic moiety, with drug-like properties.

## 1. Introduction

Pyrimidines are *N*-containing heterocyclic compounds with diverse pharmacological activities including anti-cancer [1], anti-inflammatory [2], anti-HIV [3], anti-hypertensive [4], anti-diabetic [5], and anti-microbial activity [6]. From the viewpoint of medicinal chemistry, pyrimidine derivatives have a wide variety of therapeutic applications for drug discovery because the pyrimidine scaffold is a major component of DNA and RNA [7]. In particular, many FDA-approved drugs and pharmaceuticals currently being developed contain pyrimidines as their core structure [8,9]. Therefore, pyrimidine has consistently been considered as an essential building block and a privileged scaffold for a wide range of drug candidates [8,10,11].

Calcium-activated chloride channels (CaCCs) are anion channels activated by the elevation of intracellular calcium ion concentration. Endogenous CaCCs play a role in transepithelial transport [12], the regulation of neuronal excitability and cardiomyocyte [13], sensory transduction [14,15], as well as blocking polyspermy in *Xenopus* oocyte [16].

Research on CaCCs has been conducted over the past 30 years since they were first described in *Xenopus* oocytes in the 1980s; however, their molecular identity has not yet been revealed. Identification of the CaCC gene is important for understanding its role in physiological phenomena and disease. However, gene cloning was difficult at that time because there were no specific agonists or antagonists to be used as baits. In 2008, the gene of CaCC was finally discovered by three research groups using different approaches and was named anoctamin 1 [17,18,19]. 

Anoctamin 1 (ANO1) is ubiquitously expressed in various cell types, such as non-excitable epithelial and endothelial cells, smooth muscle cells, sensory neurons, and interstitial cells of Cajal [17,20,21]. The widespread distribution of ANO1 indicates that it plays a vital role in many physiological processes, and has been implicated in the pathophysiology of various diseases such as hypertension and asthma [17,22,23]. ANO1 is also overexpressed in numerous tumor cells. In fact, ANO1 (TMEM16A) was found to be located on human chromosome 11q13, and is frequently amplified in various types of malignant tumors [24]. Because it is involved in tumorigenesis, invasion, migration, and metastasis [25,26], the development of drugs that modulate the activity of ANO1 is of great interest in cancer treatment.

**Figure 1:**
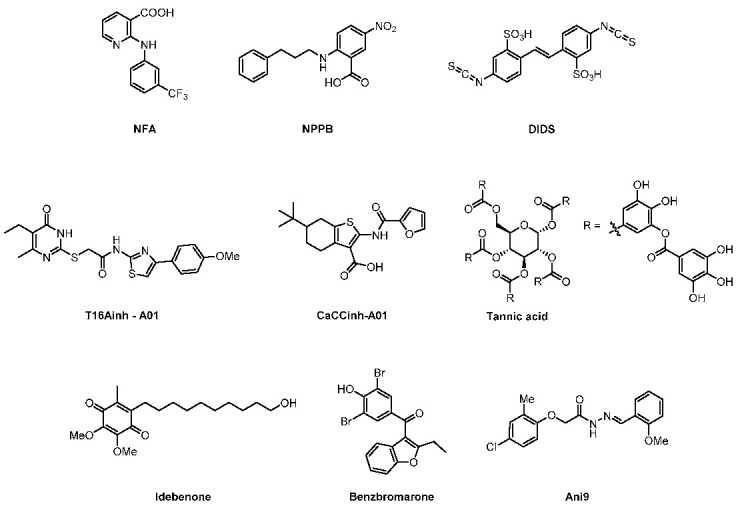
Structures of representative ANO1 channel blockers.

In previous reports, ANO1 current was effectively blocked by classical CaCC inhibitors including niflumic acid (NFA), 5-nitro-2-(3-phenylpropylamino)-benzoic acid (NPPB), and 4,4′-diisothiocyano-2,2′-stilbenedisulfonic acid (DIDS) [17]. However, these compounds lack potency and specificity, and thus, cannot be considered as drug candidates. More recently, several ANO1 inhibitors, including CaCCinh-A01 [27,28], T16Ainh-A01 [28], tannic acid [29], idebenone [30], benzbromarone [31], and Ani9 [32] have been identified through high-throughput screening (HTS) of compound libraries and were found to inhibit the proliferation of cancer cells (Figure 1). However, the information regarding the druggability and drug-likeness of these compounds is still in its infancy. For example, the benzoquinone backbone of idebenone may generate reactive oxygen species (ROS). T16Ainh-A01 and Ani9 contain potential pan-assay interferencing compounds (PAINS) structures such as heteroaryl sulfide and acylhydrazone moiety [33].

Thus, we embarked on the discovery of new structures of ANO1 channel blockers with drug-like properties. Herein, we present our work on the preparation and biological evaluation of a novel series of pyrimidine derivatives as ANO1 inhibitors and anti-cancer effects.

## 2. Results and Discussion

### 2.1. Rationale of Compound Design and Initial Library Synthesis

**Figure 2:**
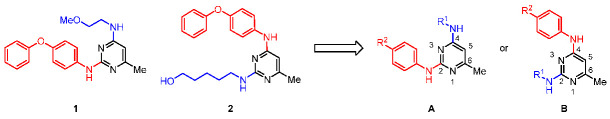
Design strategy for the construction of pyrimidine library.

Recently, a series of novel ANO1 inhibitors based on the pyrimidine scaffold was identified via screening of a focused in-house library. In the preliminary screening assay, we obtained two hit compounds **1** and **2**, containing the 2,4-disubstituted-6-methylpyrimidine scaffold with moderate inhibitory activity on ANO1 (Figure 2). The hit compounds were evaluated via an HTS campaign by halide-sensitive yellow fluorescent protein (YFP) imaging technique. Based on these results, we designed and synthesized pyrimidine analogues derived from the structures of hits **1** and **2**. Our design and synthetic strategy for novel ANO1 blockers are outlined in Figure 2. The structures of hit compounds **1** and **2** have oxygen-containing alkyl and *p*-substituted di-aryl ether substituents in common, although the substituents are oppositely established at the C2 and C4 positions in a regioisomeric pattern.

**Scheme 1:**
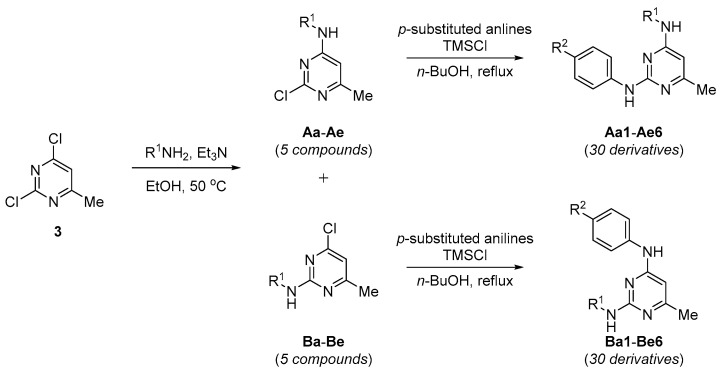
Synthetic procedure of pyrimidine library.

The construction of a focused small library of heterocyclic compounds, including pyrimidine scaffolds, commenced with the preparation of **A**- and **B**-type mono-substituted regioisomeric products as shown in Scheme 1. All 2,4-disubstituted pyrimidine derivatives were prepared via a systematic combinatorial approach in a straightforward manner. As mentioned above, hit **2** has an alkyl side chain in which the carbon length is elongated compared to that in hit **1**, although they have a *p*-substituted aniline moiety in common. Thus, we envisioned an efficient structural variation of 2,4-disubstituted pyrimidines, sequentially constructed via a two-step approach with five alkyl amines of different carbon length and six *p*-substituted anilines with diverse chemical displacements as shown in Table 1.

Nucleophilic aromatic substitution of commercially available 2,4-dichloro-6-methylpyrimidine (**3**) with five alkylamines resulted in ten mono-substituted compounds (**Aa**–**Ae** and **Ba**–**Be**, respectively), in an approximately 1:2 mixture of **C2**- and **C4**-substituted regioisomers favoring **C4**-substitution. We did not further optimize the ratio of this reaction because the initial hit compounds **1** and **2** were differently substituted at the **C2** and **C4** positions, respectively. Next, both **A**- and **B**-type regioisomeric intermediates were converted to 2,4-disubstituted pyrimidine products (**Aa1**–**Ae6** and **Ba1**–**Be6**), respectively, with six *p*-substituted anilines through a second nucleophilic aromatic substitution reaction. Aniline coupling reaction of mono-substituted pyrimidine intermediates in the presence of trimethylsilyl chloride (TMSCl) in *n*-BuOH afforded the sixty disubstituted pyrimidine products in reasonable yields (Scheme 1). The structures of synthesized compounds from **Aa1**–**Be6** are depicted in Table 1.

### 2.2. Identification of a Novel Aa3 as ANO1 Inhibitor

**Figure 3:**
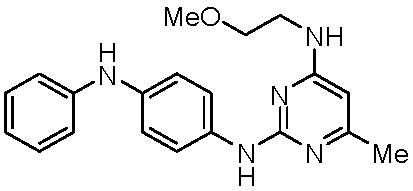
Structure of **Aa3**.

**Figure 4:**
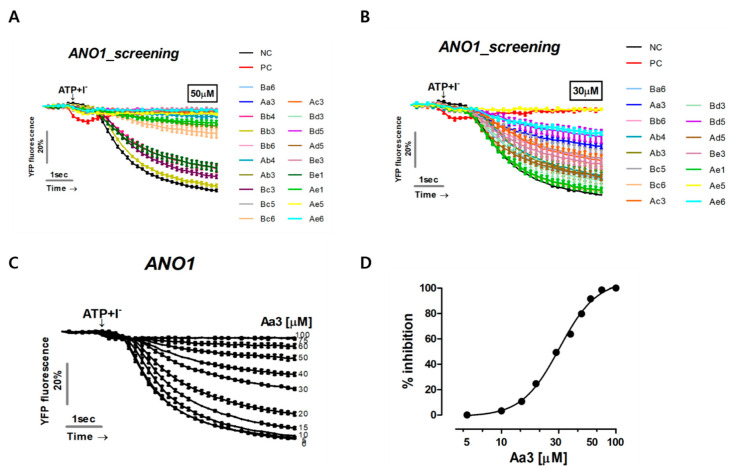
Identification of a novel inhibitor candidate of ANO1. The activity of ANO1, a Ca^2+^-activated Cl^–^ channel, was assessed in Fischer rat thyroid yellow fluorescent protein (FRT-YFP) cells stably expressing ANO1. (**A**) The cells were pre-treated with 50 µM of 19-candidate compounds for 20 min, and ANO1 was activated by treating the cells with 100 µM of ATP, an agonist of P2Y receptor. (**B**) Fifteen-compounds, which were noteworthy, were selected. Subsequently, ANO1 activity was re-measured in a lower concentration of 30 µM. (**C**) The selected **Aa3** compound inhibits the activity of ANO1 in a dose-dependent manner. (**D**) IC_50_-curve of **Aa3** (mean ± S.E. *n* = 3). NC; negative control (DMSO-treated cells), PC; positive control (cells treated with NPPB known as a chloride channel inhibitor).

To identify novel ANO1 inhibitors, a cell-based HTS assay was performed using a heterocyclic libraries containing sixty pyrimidine derivatives. As we have previously shown, ANO1 channel activity was measured by the halide-sensitive YFP imaging technique. This was done by using a cell line that stably expresses a genetically encoded iodide-sensing fluorescent protein YFP (F46L/H148Q/I152L) and ANO1 [34]. Changes in the concentration of cytoplasmic iodide alter the fluorescence intensity of the iodide-sensitive YFP; extracellular iodide influx induced by ANO1 activation leads to a decrease in fluorescence intensity. For the screening of ANO1 inhibitors, the Fischer rat thyroid (FRT) cells were plated in 96-well plates and pre-incubated with the test compounds for 20 min prior to the addition of iodide and ATP (Adenosine triphosphate), known as an agonist of P2Y2 receptor in FRT cells, for purinergic stimulation activated calcium-dependent iodide influx. After the primary screening, we selected 19 compound candidates that showed an inhibitory response of over 70% at the concentration of 100 µM (data not shown). Among them, 15 candidates exhibited the same inhibitory effect at a concentration of 50 µM (Figure 4A). The 15 selected compounds with an apparent ANO1 blocking effect were reanalyzed at a concentration of 30 µM (Figure 4B). In this evaluation step, we selected the four most potent of the 15 compound candidates. Finally, we identified a novel ANO1 inhibitor, **Aa3** (Figure 3), that inhibited ATP-induced ANO1 channel activity dose-dependently with an IC_50_ value of 32 µM (Figure 4C,D). The other three compounds such as **Bd5**, **Ae5** and **Ae6** exhibited cytotoxicity, and consequently, they were excluded from the final candidate group despite having remarkable effects (Supporting Information 16). Under physiological conditions, ANO1 is activated by various GPCR stimulation, such as purinergic receptor subtype 2 [17]. Therefore, to verify whether the **Aa3** compound directly inhibits the biological activity of ANO1 or not, we utilized A23187, a calcium ionophore, which is a mobile ion-carrier. Here, A23187 can increase intracellular Ca^2+^ concentration in intact cells by an influx of calcium from extracellular fluid across the cell membranes. As a result, we confirmed that the biological activity of ANO1 was inhibited by the **Aa3** in A23187-treated FRT-YFP-ANO cells without ATP stimulation. Therefore, these results suggest that **Aa3** can directly block the channel function of ANO1 in P2Y2 receptor-independent manner (Supporting Information 17).

### 2.3. The novel Aa3 Compound Reduces Cell Viability of ANO1 Overexpressed Cells

**Figure 5:**
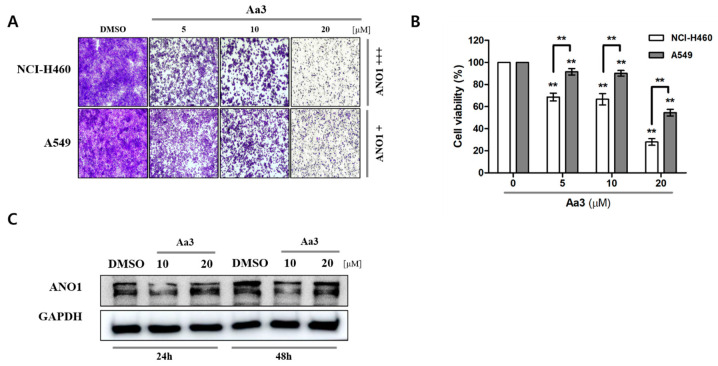
Anti-cancer effects of **Aa3** in non-small cell lung carcinoma (NSCLC) cells expressing low level of ANO1 (A549) and in those expressing high levels of ANO1 (NCI-H460). Anti-proliferation effect of **Aa3** in NCI-H460 (large cell carcinoma) and A549 (adenocarcinoma). To investigate the effects on ANO1 inhibition, NCI-H460 and A549 cells were seeded and treated with indicated concentrations of **Aa3** for 48 h. (**A**) The cells were fixed and stained using crystal violet for cell viability analysis. (**B**) Cell proliferation was analyzed using WST-1 assay and Graphpad Prism 5 software (bars, mean ± S.E.; ** *p* < 0.01, *n* > 3). (**C**) The effect on the endogenous ANO1 protein levels was examined by western blotting.

Since ANO1 has been reported to be highly expressed in many human tumors, biochemical inhibition of ANO1 activity and induction of protein degradation using selective inhibitors suggests that it can be therapeutically exploited to kill tumor cells [35]. Previous studies have revealed that several inhibitors such as CaCC_inh_-A01, T16A_inh_-A01, idebenone, *N*-((4-methoxy)-2-naphthyl)-5-nitroanthranilic acid (MONNA), Ani9, and luteolin can inhibit the proliferation of ANO1 overexpressing cancer cells [27,28,30,32,36,37,38,39,40,41,42,43,44,45,46]. To determine the biological effects of **Aa3**, we investigated whether **Aa3** could affect the proliferation of A549 and NCI-H460 cells with different expression levels of ANO1. The expression levels of ANO1 were relatively low in A549 cells than in NCI-H460 cells by the result of immunoblotting (Supporting Information 18). First, we performed the crystal violet cell cytotoxicity assay to detect cell viability. The results showed that **Aa3** remarkably reduced cell viability in a dose-dependent manner, in both NCI-H460 and A549 cells. Moreover, cell viability was significantly lower in NCI-H460 cells overexpressing ANO1 than in A549 cells (Figure 5A). Additionally, WST-1 proliferation assay showed that **Aa3** decreased proliferation of NCI-H460 cells compared with that of A549 cells (Figure 5B). Meanwhile, induction of degradation of ANO1 protein by ANO1 inhibitors could affect the growth of cancer cells [47]. To determine whether **Aa3** can induce degradation of the ANO1 protein, NCI-H460 cells was treated with **Aa3**. As shown in Figure 5C, there was no significant effect on the protein levels of endogenous ANO1 protein. These results suggest that **Aa3** can reduce proliferation or have anti-cancer effects by controlling the physiological activity of ANO1, although it cannot induce proteasomal degradation of endogenous ANO1 [1].

## 3. Materials and Methods

### 3.1. General Information

Unless otherwise noted all the starting materials and reagents were used without further purification. Thin layer chromatography was carried out using Merck silica gel 60 F_254_ plates (Merck, Kenilworth, NJ, USA), and flash chromatography was performed manually using Merck silica gel 60 (0.040–0.063 mm, 230–400 mesh, Merck, Kenilworth, NJ, USA). ^1^H- and ^13^C-NMR spectra were recorded using JEOL-500 (JEOL, Tokyo, Japan). ^1^H- and ^13^C-NMR chemical shifts are recorded in parts per million (ppm), with the residual solvent peak used as an internal reference. ^1^H-NMR data were reported in the order of chemical shift, multiplicity (brs, broad singlet; s, singlet; d, doublet; t, triplet; q, quartet; quint., quintet; m, multiplet and/or multiple resonances), number of protons, and coupling constant in Hertz (Hz). High-resolution mass spectra were obtained with Q Exactive Mass Spectrometer (Thermo Scientific, Waltham, MA, USA).

### 3.2. Representative Synthetic Procedures for Mono-Substituted Pyrimidines (***Aa***–***Ae*** and ***Ba***–***Be***)

Synthesis of **Aa** and **Ba**: To a stirred solution of 2,4-dichloro-6-methylpyrimidine (1.00 g, 6.13 mmol) in EtOH (20 mL) were added 2-methoxyethylamine (0.96 mL, 11.0 mmol, 1.8 equiv.) and triethylamine (1.71 mL, 12.3 mmol, 2.0 equiv.) at room temperature. The reaction mixture was heated to 50 °C and stirred for 4 h. The resulting mixture was cooled to room temperature and solvent was removed under reduced pressure. EtOAc (100 mL) was added to the reaction mixture and washed with water (10 mL) twice. Then organic layer was dried over MgSO_4_ and concentrated in vacuo. The residue was purified by column chromatography on silica gel (*n*-hexane/EtOAc = 3:1 to 2:1) to afford **Aa** (280 mg, 23%) and **Ba** (540 mg, 47%).

*4-Chloro-N-(2-methoxyethyl)-6-methylpyrimidin-2-amine* (**Aa**). White solid; m.p. 40–42 °C; ^1^H-NMR (500 MHz, CDCl_3_) δ 6.40 (s, 1H), 5.64 (brs, 1H), 3.57 (q, *J* = 5.3 Hz, 2H), 3.50 (t, *J* = 5.2 Hz, 2H), 3.32 (s, 3H), 2.26 (s, 3H); ^13^C-NMR (125 MHz, CDCl_3_) δ 169.6, 162.2, 161.2, 109.3, 71.2, 58.8, 41.2, 23.9; HRMS (ESI+) found 202.0747 (calculated for C_8_H_12_ClN_3_O ([M + H]^+^): 202.0742).

*2-Chloro-N-(2-methoxyethyl)-6-methylpyrimidin-4-amine* (**Ba**). Colorless oil; ^1^H-NMR (500 MHz, CDCl_3_) δ 6.09 (s, 1H), 5.38 (brs, 1H), 3.56–3.50 (m, 4H), 3.36 (s, 3H), 2.31 (s, 3H); ^13^C-NMR (125 MHz, CDCl_3_) δ 167.1, 164.1, 160.1, 103.3, 70.6, 58.8, 40.9, 23.6; HRMS (ESI+) found 202.0747 (calculated for C_8_H_12_ClN_3_O ([M + H]^+^): 202.0742).

*2-((2-chloro-6-methylpyrimidin-4-yl)amino)ethan-1-ol* (**Ab**). White solid; m.p. 130–132 °C; ^1^H-NMR (500 MHz, DMSO-*d*_6_) δ 7.73 (s, 1H), 6.25 (s, 1H), 4.71 (s, 1H), 3.46 (t, *J* = 6.0 Hz, 2H), 3.30 (brs, 2H), 2.11 (s, 3H); ^13^C-NMR (125 MHz, DMSO-*d*_6_) δ 165.4, 164.6, 159.9, 103.3, 60.2, 43.2, 23.3.

*2-((4-chloro-6-methylpyrimidin-2-yl)amino)ethan-1-ol* (**Bb**). White solid; m.p. 100–102 °C; ^1^H-NMR (500 MHz, DMSO-*d*_6_) δ 7.35 (s, 1H), 6.51 (s, 1H), 4.40 (s, 1H), 3.44 (t, *J* = 6.3 Hz, 2H), 3.27 (brs, 2H), 2.19 (s, 3H); ^13^C-NMR (125 MHz, DMSO-*d*_6_) δ 170.1, 162.5, 160.4, 108.3, 60.2, 43.9, 23.7.

*3-((2-chloro-6-methylpyrimidin-4-yl)amino)propan-1-ol* (**Ac**). White solid; m.p. 118–120 °C; ^1^H-NMR (500 MHz, DMSO-*d*_6_) δ 7.66 (t, *J* = 3.2 Hz, 1H), 6.20 (s, 1H), 4.45 (s, 1H), 3.41 (q, *J* = 5.9 Hz, 2H), 3.25–3.24 (m, 2H), 2.11 (s, 3H), 1.60 (quint., *J* = 6.7 Hz, 2H); ^13^C-NMR (125 MHz, DMSO-*d*_6_) δ 165.4, 164.4, 160.0, 103.2, 58.8, 37.7, 32.3, 23.3.

*3-((4-chloro-6-methylpyrimidin-2-yl)amino)propan-1-ol* (**Bc**). White solid; m.p. 76–78 °C; ^1^H-NMR (500 MHz, DMSO-*d*_6_) δ 7.44 (s, 1H), 6.49 (s, 1H), 4.41 (t, *J* = 5.2 Hz, 1H), 3.40 (q, *J* = 5.9 Hz, 2H), 3.24 (brs, 2H), 2.19 (s, 3H), 1.61 (quint., *J* = 6.7 Hz, 2H); ^13^C-NMR (125 MHz, DMSO-*d*_6_) δ 170.1, 162.5, 160.3, 108.1, 59.0, 38.5, 32.4, 23.7.

*4-((2-chloro-6-methylpyrimidin-4-yl)amino)butan-1-ol* (**Ad**). White solid; m.p. 82–84 °C; ^1^H-NMR (500 MHz, DMSO-*d*_6_) δ 7.68 (s, 1H), 6.19 (s, 1H), 4.38 (t, *J* = 4.9 Hz, 1H), 3.36 (q, *J* = 5.7 Hz, 2H), 3.21–3.20 (m, 2H), 2.11 (s, 3H), 1.48 (quint., *J* = 7.0 Hz, 2H), 1.41 (quint., *J* = 6.6 Hz, 2H); ^13^C-NMR (125 MHz, DMSO-*d*_6_) δ 165.4, 164.4, 160.0, 103.1, 60.9, 41.7, 30.3, 25.8, 23.3.

*4-((4-chloro-6-methylpyrimidin-2-yl)amino)butan-1-ol* (**Bd**). White solid; m.p. 68–70 °C; ^1^H-NMR (500 MHz, DMSO-*d*_6_) δ 7.47 (s, 1H), 6.47 (s, 1H), 4.34 (t, *J* = 4.9 Hz, 1H), 3.36 (q, *J* = 5.9 Hz, 2H), 3.19 (brs, 2H), 2.18 (s, 3H), 1.48 (quint., *J* = 7.0 Hz, 2H), 1.40 (quint., *J* = 6.9 Hz, 2H); ^13^C-NMR (125 MHz, DMSO-*d*_6_) δ 170.1, 162.6, 160.4, 108.1, 61.2, 41.0, 30.4, 25.9, 23.7.

*5-((2-chloro-6-methylpyrimidin-4-yl)amino)pentan-1-ol* (**Ae**). White solid; m.p. 88–90 °C; ^1^H-NMR (500 MHz, DMSO-*d*_6_) δ 7.67 (s, 1H), 6.19 (s, 1H), 4.32 (t, *J* = 4.9 Hz, 1H), 3.34 (q, *J* = 5.9 Hz, 2H), 3.19 (q, *J* = 5.2 Hz, 2H), 2.11 (s, 3H), 1.45 (quint., *J* = 7.3 Hz, 2H), 1.39 (quint., *J* = 7.2 Hz, 2H), 1.45 (quint., *J* = 7.6 Hz, 2H); ^13^C-NMR (125 MHz, DMSO-*d*_6_) δ 165.3, 164.4, 160.0, 103.1, 61.1, 41.4, 32.6, 28.9, 23.4. 

*5-((4-chloro-6-methylpyrimidin-2-yl)amino)pentan-1-ol* (**Be**). White solid; m.p. 128–130 °C; ^1^H-NMR (500 MHz, DMSO-*d*_6_) δ 7.47 (s, 1H), 6.48 (s, 1H), 4.30 (t, *J* = 4.9 Hz, 1H), 3.34 (q, *J* = 5.7 Hz, 2H), 3.17 (brs, 2H), 2.18 (s, 3H), 1.45 (quint., *J* = 7.3 Hz, 2H), 1.39 (quint., *J* = 7.0 Hz, 2H), 1.25 (quint., *J* = 7.6 Hz, 2H); ^13^C-NMR (125 MHz, DMSO-*d*_6_) δ 170.1, 162.6, 160.2, 108.1, 61.1, 41.1, 32.7, 29.1, 23.7, 23.2.

### 3.3. Representative Synthetic Procedures for Di-Substituted Pyrimidines (***Aa1***–***Ae6*** and ***Ba1***–***Be6***)

To a stirred solution of **Aa** (100 mg, 0.496 mmol) and 4-aminodiphenylamine (183 mg, 0.992 mmol, 2.0 equiv.) in *n*-BuOH (4 mL) was added chlrotrimethylsilane (5 drops) at room temperature. The reaction mixture was heated to reflux and stirred overnight. The resulting mixture was cooled to room temperature and the solvent (*n*-BuOH) was removed under reduced pressure. The crude product was purified by column chromatography on silica gel (DCM/MeOH = 20:1 to 10:1) to afford **Aa3** (280 mg, 81%).

*N^2^-(4-isopropoxyphenyl)-N^4^-(2-methoxyethyl)-6-methylpyrimidine-2,4-diamine* (**Aa1**). Yield: 75%; pale brown oil; ^1^H-NMR (500 MHz, DMSO-*d*_6_) δ 8.68 (s, 1H), 7.59 (d, *J* = 8.6 Hz, 2H), 6.70 (s, 1H), 6.73 (dt, *J* = 8.6, 2.6 Hz, 2H), 5.74 (s, 1H), 4.44 (quint., *J* = 6.0 Hz, 1H), 3.42 (brs, 4H), 3.23 (s, 3H), 2.05 (s, 3H), 1.19 (d, *J* = 6.3 Hz, 6H); ^13^C-NMR (125 MHz, DMSO-*d*_6_) δ 163.7, 160.0, 151.9, 135.2, 120.3, 116.2, 95.7, 71.1, 69.9, 58.5, 40.3, 23.8, 22.4; HRMS (ESI+) found 317.1978 (calculated for C_17_H_25_N_4_O_2_ ([M + H]^+^): 317.1968).

*N^4^-(2-methoxyethyl)-6-methyl-N^2^-(4-morpholinophenyl)pyrimidine-2,4-diamine* (**Aa2**). Yield: 54%; dark brown solid; m.p. 195–197 °C; ^1^H-NMR (500 MHz, DMSO-*d*_6_) δ 8.65 (s, 1H), 7.61 (d, *J* = 9.2 Hz, 2H), 6.96 (brs, 1H), 6.78 (d, *J* = 9.2 Hz, 2H), 5.75 (s, 1H), 3.67 (t, *J* = 4.6 Hz, 4H), 3.43 (m, 4H), 3.23 (d, *J* = 11.4 Hz, 3H), 2.94 (t, *J* = 4.6 Hz, 2H), 2.06 (s, 3H); ^13^C-NMR (125 MHz, DMSO-*d*_6_) δ 163.7, 160.1, 145.8, 134.7, 120.0, 116.1, 95.7, 71.2, 66.7, 58.5, 50.0, 40.3, 23.9; HRMS (ESI+) found 344.2087 (calculated for C_18_H_26_N_5_O_2_ ([M + H]^+^): 344.2084). 

*N^4^-(2-methoxyethyl)-6-methyl-N^2^-(4-(phenylamino)phenyl)pyrimidine-2,4-diamine* (**Aa3**). Yield: 81%; blue solid; m.p. 143–145 °C; ^1^H-NMR (500 MHz, CDCl_3_) δ 7.50 (d, *J* = 8.6 Hz, 2H), 7.21 (t, *J* = 7.7 Hz, 2H), 7.04–7.03 (m, 3H), 6.96 (d, *J* = 7.5 Hz, 2H), 6.83 (t, *J* = 7.5 Hz, 1H), 5.70 (s, 1H), 5.64 (s, 1H), 5.16 (brs, 1H), 3.56-3.51 (m, 4H), 3.37 (s, 3H), 2.23 (s, 3H); ^13^C-NMR (125 MHz, CDCl_3_) δ 165.4, 163.5, 159.7, 144.6, 137.0, 134.9, 129.3, 120.5, 120.4, 119.8, 116.2, 94.4, 71.1, 58.9, 40.9, 23.8; HRMS (ESI+) found 350.1981 (calculated for C_20_H_24_N_5_O ([M + H]^+^): 350.1971).

*N^4^-(2-methoxyethyl)-6-methyl-N^2^-(4-phenoxyphenyl)pyrimidine-2,4-diamine* (**Aa4**). Yield: 81%; white solid; m.p. 159–160 °C; ^1^H-NMR (500 MHz, DMSO-*d*_6_) δ 8.93 (s, 1H), 7.78 (d, *J* = 9.2 Hz, 2H), 7.28 (dd, *J* = 8.6, 7.5 Hz, 2H), 7.02-6.99 (m, 2H), 6.96-6.83 (m, 4H), 5.80 (s, 1H), 3.43 (brs, 4H), 3.22 (s, 3H), 2.08 (s, 3H); ^13^C-NMR (125 MHz, DMSO-*d*_6_) δ 163.7, 160.0, 158.6, 149.7, 138.4, 130.3, 122.9, 120.2, 120.0, 117.8, 96.0, 71.1, 58.5, 40.3, 23.9; HRMS (ESI+) found 351.1821 (calculated for C_20_H_23_N_4_O_2_ ([M + H]^+^): 351.1811).

*N^2^-(4-(4-chlorophenoxy)phenyl)-N^4^-(2-methoxyethyl)-6-methylpyrimidine-2,4-diamine* (**Aa5**). Yield: 72%; white solid; m.p. 160–161 °C; ^1^H-NMR (500 MHz, DMSO-*d*_6_) δ 8.97 (s, 1H), 7.50 (dt, *J* = 9.2, 2.3 Hz, 2H), 7.21 (dt, *J* = 9.2, 2.9 Hz, 2H), 7.04 (brs, 1H), 6.96-6.83 (m, 4H), 5.81 (s, 1H), 4.43 (brs, 4H), 3.22 (s, 3H), 2.08 (s, 3H); ^13^C-NMR (125 MHz, DMSO-*d*_6_) δ 163.7, 160.0, 157.6, 149.2, 138.7, 130.1, 126.6, 120.2, 119.2, 96.1, 71.1, 58.4, 40.3, 23.9; HRMS (ESI+) found 385.1431 (calculated for C_20_H_22_ClN_4_O_2_ ([M + H]^+^): 385.1422).

*N^4^-(2-methoxyethyl)-6-methyl-N^2^-(4-(p-tolyloxy)phenyl)pyrimidine-2,4-diamine* (**Aa6**). Yield: 79%; white solid; m.p. 128–129 °C; ^1^H-NMR (500 MHz, DMSO-*d*_6_) δ 8.90 (s, 1H), 7.75 (d, *J* = 9.2, 2.9 Hz, 2H), 7.09 (d, *J* = 8.6 Hz, 2H), 7.03 (brs, 1H), 6.84 (dt, *J* = 8.6, 2.6 Hz, 2H), 6.80 (dt, *J* = 8.5, 2.6 Hz, 2H), 5.79 (s, 1H), 3.43 (brs, 4H), 3.22 (s, 3H), 2.21 (s, 3H), 2.07 (s, 3H); ^13^C-NMR (125 MHz, DMSO-*d*_6_) δ 163.7, 160.0, 156.1, 150.3, 138.0, 131.9, 130.6, 120.2, 119.5, 118.0, 96.1, 71.1, 58.4, 40.2, 23.9, 20.7; HRMS (ESI+) found 365.1967 (calculated for C_20_H_23_N_4_O_2_ ([M + H]^+^): 365.1978).

*N^4^-(4-isopropoxyphenyl)-N^2^-(2-methoxyethyl)-6-methylpyrimidine-2,4-diamine* (**Ba1**). Yield: 71%; black solid; m.p. 140–142 °C; ^1^H-NMR (500 MHz, DMSO-*d*_6_) δ 8.80 (s, 1H), 7.49 (d, *J* = 8.6 Hz, 2H), 6.78 (dt, *J* = 8.6, 2.6 Hz, 2H), 6.44 (s, 1H), 5.77 (s, 1H), 4.44 (quint., *J* = 6.0 Hz, 1H), 3.42–3.36 (m, 4H), 3.22 (s, 3H), 2.04 (s, 3H), 1.19 (d, *J* = 6.3 Hz, 6H); ^13^C-NMR (125 MHz, DMSO-*d*_6_) δ 164.9, 162.2, 161.7, 152.8, 134.1, 121.8, 116.4, 94.6, 71.4, 69.9, 58.4, 40.7, 24.0, 22.4; HRMS (ESI+) found 317.1978 (calculated for C_17_H_24_N_4_O_2_ ([M + H]^+^): 317.1968).

*N^2^-(2-methoxyethyl)-6-methyl-N^4^-(4-morpholinophenyl)pyrimidine-2,4-diamine* (**Ba2**). Yield: 60%; dark blue solid; m.p. 124–126 °C; ^1^H-NMR (500 MHz, DMSO-*d*_6_) δ 8.80 (brs, 1H), 7.46 (d, *J* = 8.6 Hz, 2H), 6.82 (d, *J* = 9.2 Hz, 2H), 6.44 (brs, 1H), 5.76 (s, 1H), 3.68 (t, *J* = 4.6 Hz, 4H), 3.41-3.36 (m, 4H), 3.22 (s, 3H), 2.98 (t, *J* = 4.6 Hz, 4H), 2.04 (s, 3H); ^13^C-NMR (125 MHz, DMSO-*d*_6_) δ 164.6, 162.2, 161.8, 146.8, 133.4, 121.5, 116.1, 94.7, 71.3, 66.7, 58.4, 49.7, 40.7, 23.9; HRMS (ESI+) found 344.2087 (calculated for C_18_H_26_N_5_O_2_ ([M + H]^+^): 344.2077).

*N^2^-(2-methoxyethyl)-6-methyl-N^4^-(4-(phenylamino)phenyl)pyrimidine-2,4-diamine* (**Ba3**). Yield: 59%; pale purple foam; ^1^H-NMR (500 MHz, DMSO-*d*_6_) δ 8.79 (s, 1H), 7.90 (s, 1H), 7.49 (d, *J* = 8.1 Hz, 2H), 7.21 (dd, *J* = 8.6, 7.5 Hz, 2H), 6.97 (d, *J* = 8.6 Hz, 2H), 6.94 (d, *J* = 7.5 Hz, 2H), 6.70 (t, *J* = 7.2 Hz, 1H), 6.42 (brs, 1H), 5.76 (s, 1H), 3.45–3.45 (m, 4H), 3.21 (s, 3H), 2.04 (s, 3H); ^13^C-NMR (125 MHz, DMSO-*d*_6_) δ 164.5, 162.0, 161.7, 144.9, 138.0, 134.1, 129.6, 121.6, 119.2, 118.8, 116.0, 95.1, 71.3, 58.4, 40.7, 23.9; HRMS (ESI+) found 350.1981 (calculated for C_20_H_24_N_5_O ([M + H]^+^): 350.1968). 

*N^2^-(2-methoxyethyl)-6-methyl-N^4^-(4-phenoxyphenyl)pyrimidine-2,4-diamine* (**Ba4**). Yield: 70%; white solid; m.p. 145–146 °C; ^1^H-NMR (500 MHz, DMSO-*d*_6_) δ 9.06 (s, 1H), 7.70 (d, *J* = 8.6 Hz, 2H), 7.30 (t, *J* = 8.0 Hz, 2H), 7.03 (t, *J* = 7.5 Hz, 1H), 6.92 (d, *J* = 9.2 Hz, 4H), 6.54 (brs, 1H), 5.85 (s, 1H), 3.43-3.38 (m, 4H), 3.20 (s, 3H), 2.07 (s, 3H); ^13^C-NMR (125 MHz, DMSO-*d*_6_) δ 165.2, 162.2, 161.5, 158.2, 150.8, 137.3, 130.3, 123.2, 121.4, 120.0, 118.1, 95.3, 71.3, 58.4, 40.8, 24.1; HRMS (ESI+) found 351.1821 (calculated for C_20_H_23_N_4_O_2_ ([M + H]^+^): 351.1811).

*N^4^-(4-(4-chlorophenoxy)phenyl)-N^2^-(2-methoxyethyl)-6-methylpyrimidine-2,4-diamine* (**Ba5**). Yield: 74%; white solid; m.p. 143–144 °C; ^1^H-NMR (500 MHz, DMSO-*d*_6_) δ 9.05 (s, 1H) 7.70 (d, *J* = 8.6 Hz, 2H), 7.34 (dt, *J* = 9.2, 2.9 Hz, 2H), 6.96-6.83 (m, 4H), 6.53 (s, 1H), 5.83 (s, 1H), 3.43-3.34 (m, 4H), 3.20 (s, 3H), 2.07 (s, 3H); ^13^C-NMR (125 MHz, DMSO-*d*_6_) δ 165.4, 162.3, 161.5, 157.2, 150.2, 137.8, 130.2, 126.3, 121.3, 120.2, 119.6, 95.1, 71.3, 58.4, 40.8, 24.1; HRMS (ESI+) found 385.1431 (calculated for C_20_H_21_ClN_4_O_2_ ([M + H]^+^): 385.1422).

*N^2^-(2-methoxyethyl)-6-methyl-N^4^-(4-(p-tolyloxy)phenyl)pyrimidine-2,4-diamine* (**Ba6**). Yield: 77%; Gray solid; m.p. 140–142 °C; ^1^H-NMR (500 MHz, DMSO-*d*_6_) δ 8.96 (s, 1H), 7.64 (d, *J* = 8.6 Hz, 2H), 7.11 (d, *J* = 8.6 Hz, 2H), 6.86 (dt, *J* = 9.2, 2.9 Hz, 2H), 6.82 (dt, *J* = 8.6, 2.9 Hz, 2H), 6.48 (s, 1H), 5.80 (s, 1H), 3.42-3.29 (m, 4H), 3.20 (s, 3H), 2.23 (s, 3H), 2.05 (s, 3H); ^13^C-NMR (125 MHz, DMSO-*d*_6_) δ 162.7, 161.5, 155.8, 151.4, 137.0, 132.3, 130.7, 121.3, 119.4, 118.4, 71.3, 58.4, 40.8, 23.0, 20.7; HRMS (ESI+) found 365.1978 (calculated for C_21_H_24_N_4_O_2_ ([M + H]^+^): 365.1969).

*2-((2-((4-isopropoxyphenyl)amino)-6-methylpyrimidin-4-yl)amino)ethan-1-ol* (**Ab1**). White solid; ^1^H-NMR (500 MHz, DMSO-*d*_6_) δ 8.95 (s, 1H), 8.71 (s,1H), 7.61 (d, *J* = 9.5 Hz, 2H), 7.00 (brs, 1H), 6.74 (d, *J* = 8.6 Hz, 2H), 5.76 (s, 1H), 4.72 (s, 1H), 4.42 (heptet, *J* = 6.0 Hz, 1H), 3.52 (t, *J* = 5.5 Hz, 2H), 3.33 (brs, 2H), 2.07 (s, 3H), 1.18 (d, *J* = 5.8 Hz, 6H); ^13^C-NMR (125 MHz, CDCl_3_) δ 163.8, 159.8, 152.0, 135.1, 120.4, 116.3, 95.7, 69.9, 60.3, 43.5, 23.7, 22.4.

*2-((6-methyl-2-((4-morpholinophenyl)amino)pyrimidin-4-yl)amino)ethan-1-ol* (**Ab2**). Purple solid; m.p. 206–208 °C; ^1^H-NMR (500 MHz, DMSO-*d*_6_) δ 10.10 (s, 1H), 8.94 (s, 1H), 7.38 (d, *J* = 8.0 Hz, 2H), 6.91 (d, *J* = 8.1 Hz, 2H), 5.99 (s, 1H), 4.89 (brs, 1H), 3.69 (brs, 4H), 3.52 (brs, 2H), 3.39 (brs, 2H), 3.03 (brs, 4H), 2.18 (s, 3H); ^13^C-NMR (125 MHz, DMSO-*d*_6_) δ 163.3, 153.0, 152.0, 148.4, 129.4, 122.3, 115.9, 96.9, 66.6, 59.4, 49.1, 44.1, 18.9.

*2-((6-methyl-2-((4-(phenylamino)phenyl)amino)pyrimidin-4-yl)amino)ethan-1-ol* (**Ab3**). Purple solid; ^1^H-NMR (500 MHz, DMSO-*d*_6_) δ 10.25 (s, 1H), 9.14 (s, 1H), 8.26 (s, 1H), 7.37 (d, *J* = 8.6 Hz, 2H), 7.17 (t, *J* = 7.8 Hz, 2H), 7.06 (t, *J* = 8.6 Hz, 2H), 7.02 (d, *J* = 8.0 Hz, 2H), 6.75 (t, *J* = 7.2 Hz, 1H), 6.02 (s, 1H), 4.94 (brs, 1H), 3.53 (t, *J* = 5.5 Hz, 2H), 3.40 (q, *J* = 5.4 Hz, 2H), 2.19 (s, 3H); ^13^C-NMR (125 MHz, DMSO-*d*_6_) δ 163.3, 152.7, 151.4, 144.0, 140.6, 129.6, 122.5, 119.9, 117.7, 116.9, 96.9, 59.4, 44.2, 18.7.

*2-((6-methyl-2-((4-phenoxyphenyl)amino)pyrimidin-4-yl)amino)ethan-1-ol* (**Ab4**). White solid; m.p. 245–247 °C; ^1^H-NMR (500 MHz, DMSO-*d*_6_) δ 10.30 (s, 1H), 8.97 (s, 1H), 7.64 (d, *J* = 6.3 Hz, 2H), 7.34 (t, *J* = 7.2 Hz, 2H), 7.09 (t, *J* = 6.9 Hz, 1H), 7.00 (d, *J* = 8.1 Hz, 2H), 6.97 (d, *J* = 7.5 Hz, 2H), 6.04 (s, 1H), 4.87 (brs, 1H), 3.52 (brs, 2H), 3.40 (brs, 2H), 2.21 (s, 3H); ^13^C-NMR (125 MHz, DMSO-*d*_6_) δ 163.3, 157.5, 153.3, 153.1, 133.4, 130.5, 123.8, 122.8, 119.8, 118.8, 97.3, 59.4, 44.2, 19.0.

*2-((2-((4-(4-chlorophenoxy)phenyl)amino)-6-methylpyrimidin-4-yl)amino)ethan-1-ol* (**Ab5**). White solid; m.p. 244–246 °C; ^1^H-NMR (500 MHz, DMSO-*d*_6_) δ 8.96 (s, 1H), 7.80 (d, *J* = 9.2 Hz, 2H), 7.33 (dt, *J* = 9.2, 2.9 Hz, 2H), 7.01 (brs, 1H), 6.90 (d, *J* = 8.6 Hz, 4H), 5.79 (s, 1H), 4.68 (brs, 1H), 3.51 (t, *J* = 5.7 Hz, 2H), 4.33 (brs, 2H), 2.08 (s, 3H); ^13^C-NMR (125 MHz, CDCl_3_) δ 163.8, 159.9, 157.6, 149.2, 138.7, 130.1, 126.5, 120.2, 120.2, 119.3, 96.1, 60.3, 43.6, 23.8.

*2-((6-methyl-2-((4-(p-tolyloxy)phenyl)amino)pyrimidin-4-yl)amino)ethan-1-ol* (**Ab6**). Gray solid; ^1^H-NMR (500 MHz, DMSO-*d*_6_) δ 8.91 (s, 1H), 7.77 (d, *J* = 8.6 Hz, 2H), 7.08 (d, *J* = 8.1 Hz, 2H), 7.01 (brs, 1H), 6.85 (d, *J* = 9.2 Hz, 2H), 6.80 (d, *J* = 8.0 Hz, 2H), 5.80 (s, 1H), 4.71 (brs, 1H), 3.52 (t, *J* = 5.7 Hz, 2H), 3.34 (brs, 2H), 2.21 (s, 3H), 2.08 (s, 3H); ^13^C-NMR (125 MHz, CDCl_3_) δ 163.8, 159.9, 156.1, 150.3, 138.0, 132.0, 130.6, 120.2, 119.5, 118.0, 96.3, 60.3, 43.4, 23.8, 20.6.

*2-((4-((4-isopropoxyphenyl)amino)-6-methylpyrimidin-2-yl)amino)ethan-1-ol* (**Bb1**). Gray solid; m.p. 214–216 °C; ^1^H-NMR (500 MHz, DMSO-*d*_6_) δ 10.53 (s, 1H), 7.54 (m, 3H), 6.89 (d, *J* = 9.2 Hz, 2H), 6.05 (s, 1H), 4.89 (s, 1H), 4.45 (heptet, *J* = 6.0 Hz, 1H), 3.52 (t, *J* = 5.5 Hz, 2H), 3.39 (q, *J* = 5.7 Hz, 2H), 2.23 (s, 3H), 1.22 (d, *J* = 5.8 Hz, 6H); ^13^C-NMR (125 MHz, CDCl_3_) δ 160.9, 154.9, 154.7, 152.5, 131.3, 123.2, 116.2, 96.9, 69.9, 59.5, 44.0, 22.3, 18.8.

*2-((4-methyl-6-((4-morpholinophenyl)amino)pyrimidin-2-yl)amino)ethan-1-ol* (**Bb2**). White solid; ^1^H-NMR (500 MHz, DMSO-*d*_6_) δ 10.75 (s, 1H), 7.72 (s, 1H), 7.57 (d, *J* = 8.1 Hz, 2H), 6.91 (d, *J* = 8.6 Hz, 2H), 6.13 (s, 1H), 4.90 (brs, 1H), 3.69 (t, *J* = 4.6 Hz, 4H), 3.52 (t, *J* = 5.7 Hz, 2H), 3.40 (q, *J* = 5.2 Hz, 2H), 3.05 (m, 4H), 2.21 (s, 3H); ^13^C-NMR (125 MHz, DMSO-*d*_6_) δ 160.8, 154.9, 152.2, 148.3, 130.4, 122.5, 115.6, 97.1, 66.5, 59.5, 49.0, 43.8, 18.8.

*N^2^-(2-methoxyethyl)-6-methyl-N^4^-(4-(phenylamino)phenyl)pyrimidine-2,4-diamine* (**Bb3**). Blue solid; m.p. 228–230 °C; ^1^H-NMR (500 MHz, DMSO-*d*_6_) δ 10.63 (s, 1H), 8.22 (s, 1H), 7.66–7.53 (m, 2H), 7.19 (t, *J* = 7.2 Hz, 2H), 7.05 (t, *J* = 9.2 Hz, 2H), 7.03 (d, *J* = 8.0 Hz, 2H), 6.77 (t, *J* = 7.2 Hz, 1H), 6.08 (s, 1H), 4.89 (brs, 1H), 3.52 (t, *J* = 5.7 Hz, 2H), 3.40 (q, *J* = 5.3 Hz, 2H), 2.22 (s, 3H); ^13^C-NMR (125 MHz, DMSO-*d*_6_) δ 160.6, 154.9, 152.0, 143.9, 140.7, 130.8, 129.6, 122.8, 120.1, 117.3, 117.1, 96.9, 59.5, 43.8, 18.8.

*2-((4-methyl-6-((4-phenoxyphenyl)amino)pyrimidin-2-yl)amino)ethan-1-ol* (**Bb4**). Gray solid; ^1^H-NMR (500 MHz, DMSO-*d*_6_) δ 10.36 (s, 1H), 9.07 (s, 1H), 7.54 (d, *J* = 9.2 Hz, 2H), 7.35 (t, *J* = 8.0 Hz, 2H), 7.09 (t, *J* = 7.4 Hz, 1H), 7.01 (d, *J* = 9.2 Hz, 2H), 6.99 (d, *J* = 9.2 Hz, 2H), 6.05 (s, 1H), 4.80 (brs, 1H), 3.52 (t, *J* = 5.4 Hz, 2H), 3.40 (q, *J* = 5.4 Hz, 2H), 2.22 (s, 3H); ^13^C-NMR (125 MHz, CDCl_3_) δ 163.2, 157.4, 153.4, 152.7, 151.7, 133.2, 130.5, 123.9, 123.0, 119.8, 118.9, 97.4, 59.4, 44.2, 18.8.

*2-((4-((4-(4-chlorophenoxy)phenyl)amino)-6-methylpyrimidin-2-yl)amino)ethan-1-ol* (**Bb5**). Gray solid; ^1^H-NMR (500 MHz, DMSO-*d*_6_) δ 9.11 (s, 1H), 7.72 (d, *J* = 8.6 Hz, 2H), 7.34 (dt, *J* = 9.2, 2.9 Hz, 2H), 6.96–6.92 (m, 4H), 6.52 (s, 1H), 5.85 (s, 1H), 4.64 (brs, 1H), 3.49 (t, *J* = 6.0 Hz, 2H), 3.31 (q, *J* = 6.1 Hz, 2H), 2.07 (s, 3H); ^13^C-NMR (125 MHz, CDCl_3_) δ 164.9, 162.2, 161.5, 157.2, 150.3, 137.7, 130.2, 126.9, 121.4, 120.2, 119.6, 95.1, 60.7, 44.1, 24.0.

*N^2^-(2-methoxyethyl)-6-methyl-N^4^-(4-(p-tolyloxy)phenyl)pyrimidine-2,4-diamine* (**Bb6**). Gray solid; m.p. 108–110 °C; ^1^H-NMR (500 MHz, DMSO-*d*_6_) δ 10.77 (s, 1H), 7.69 (brs, 3H), 7.16 (d, *J* = 8.0 Hz, 2H), 6.95 (d, *J* = 8.6 Hz, 2H), 6.89 (dt, *J* = 8.6 Hz, 2H), 6.13 (s, 1H), 4.88 (brs, 1H), 3.51 (t, *J* = 5.5 Hz, 2H), 3.40 (q, *J* = 5.2 Hz, 2H), 2.49 (s, 6H); ^13^C-NMR (125 MHz, CDCl_3_) δ 161.3, 154.9, 154.7, 154.2, 152.9, 133.8, 133.2, 130.9, 123.2, 119.3, 118.9, 97.1, 59.5, 43.8, 20.7, 18.9.

*3-((2-((4-isopropoxyphenyl)amino)-6-methylpyrimidin-4-yl)amino)propan-1-ol* (**Ac1**). Gray solid; m.p. 156–158 °C; ^1^H-NMR (500 MHz, DMSO-*d*_6_) δ 10.19 (s, 1H), 9.1 (s, 1H), 7.42 (d, *J* = 9.6 Hz, 2H), 6.88 (d, *J* = 9.2 Hz, 2H), 5.98 (s, 1H), 4.54 (s, 1H), 4.52 (heptet, *J* = 6.0 Hz, 1H), 3.41 (t, *J* = 6.0 Hz, 2H), 3.36 (q, *J* = 6.3 Hz, 2H), 2.19 (s, 2H), 1.65 (quint., *J* = 6.6 Hz, 2H), 1.21 (d, *J* = 6.3 Hz, 6H); ^13^C-NMR (125 MHz, DMSO-*d*_6_) δ 161.2, 155.6, 154.6, 131.6, 123.2, 116.2, 96.2, 69.9, 58.8, 38.6, 32.3, 22.3, 19.4.

*2-((6-methyl-2-((4-morpholinophenyl)amino)pyrimidin-4-yl)amino)propan-1-ol* (**Ac2**). Black solid; m.p. 135–137 °C; ^1^H-NMR (500 MHz, DMSO-*d*_6_) δ 9.04 (s, 1H), 7.54 (d, *J* = 7.5 Hz, 2H), 6.82 (d, *J* = 8.0 Hz, 2H), 5.77 (s, 1H), 4.47 (s, 1H), 3.68 (brs, 4H), 3.43 (s, 2H), 3.33 (s, 2H), 2.97 (s, 3H), 2.08 (s, 3H), 1.64 (quint., *J* = 6.6 Hz, 2H); ^13^C-NMR (125 MHz, DMSO-*d*_6_) δ 163.6, 146.5, 133.2, 120.7, 116.1, 95.6, 66.7, 58.9, 49.8, 38.0, 32.6, 22.3.

*2-((6-methyl-2-((4-(phenylamino)phenyl)amino)pyrimidin-4-yl)amino)propan-1-ol* (**Ac3**). Blue solid; m.p. 113–115 °C; ^1^H-NMR (500 MHz, DMSO-*d_6_*) δ 9.59 (s, 1H), 8.25 (s, 1H), 8.04 (s, 1H), 7.50 (d, *J* = 8.6 Hz, 2H), 7.14 (t, *J* = 8.0 Hz, 2H), 7.02 (d, *J* = 8.0 Hz, 2H), 6.71 (t, *J* = 7.2 Hz, 2H), 5.89 (s, 1H), 4.53 (s, 1H), 3.44 (d, *J* = 6.3 Hz, 2H), 3.36-3.33 (m, 2H), 2.14 (s, 3H), 1.66 (quint., *J* = 6.6 Hz, 2H); ^13^C-NMR (125 MHz, DMSO-*d*_6_) δ 163.3, 155.8, 144.7, 138.9, 131.9, 129.6, 121.7, 119.4, 118.4, 116.2, 96.5, 58.8, 38.3, 32.4, 20.6.

*2-((6-methyl-2-((4-phenoxyphenyl)amino)pyrimidin-4-yl)amino)propan-1-ol* (**Ac4**). White solid; ^1^H-NMR (500 MHz, DMSO-*d*_6_) δ 8.94 (s, 1H), 7.81 (dt, *J* = 9.2, 2.6 Hz, 2H), 7.28 (m, 2H), 7.00 (t, *J* = 7.5 Hz, 1H), 6.89 (d, *J* = 9.2 Hz, 4H), 5.76 (s, 1H), 4.47 (brs, 1H), 3.45 (t, *J* = 6.3 Hz, 2H), 3.32 (brs, 2H), 2.08 (s, 3H), 1.67 (quint., *J* = 6.7 Hz, 2H); ^13^C-NMR (125 MHz, DMSO-*d*_6_) δ 163.7, 159.9, 158.6, 149.6, 138.4, 130.3, 122.8, 120.2, 120.1, 117.7, 95.8, 59.1, 37.9, 32.8, 23.8.

*2-((2-((4-(4-chlorophenoxy)phenyl)amino)-6-methylpyrimidin-4-yl)amino)propan-1-ol* (**Ac5**). White solid; ^1^H-NMR (500 MHz, DMSO-*d*_6_) δ 8.96 (s, 1H), 7.83 (dt, *J* = 9.2, 2.6 Hz, 2H), 7.32 (dt, *J* = 8.6, 2.9 Hz, 2H), 6.88 (dt, *J* = 9.2, 2.6 Hz, 2H), 6.98 (brs, 1H), 6.92-6.88 (m, 4H), 5.76 (s, 1H), 4.47 (s, 1H), 3.46 (t, *J* = 6.0 Hz, 2H), 3.32 (brs, 2H), 2.08 (s, 3H), 1.67 (quint., *J* = 6.6 Hz, 2H); ^13^C-NMR (125 MHz, DMSO-*d*_6_) δ 163.7, 159.9, 157.6, 149.1, 138.8, 130.1, 126.5, 120.2, 120.2, 119.2, 96.6, 59.1, 37.8, 32.8, 23.8.

*2-((6-methyl-2-((4-(p-tolyloxy)phenyl)amino)pyrimidin-4-yl)amino)propan-1-ol* (**Ac6**). White solid; ^1^H-NMR (500 MHz, DMSO-*d*_6_) δ 10.37 (s, 1H), 9.05 (s, 1H), 7.56 (d, *J* = 8.6 Hz, 2H), 7.14 (d, *J* = 8.0 Hz, 2H), 6.95 (d, *J* = 9.2 Hz, 2H), 6.86 (t, *J* = 8.0 Hz, 2H), 6.01 (s, 1H), 4.54 (s, 1H), 3.41 (t, *J* = 6.0 Hz, 2H), 3.38 (q, *J* = 6.3 Hz, 2H), 2.23 (s, 3H), 2.20 (s, 1H), 1.66 (quint., *J* = 6.6 Hz, 2H); ^13^C-NMR (125 MHz, DMSO-*d*_6_) δ 163.0, 155.0, 153.7, 152.9, 151.9, 133.1, 132.9, 130.9, 122.7, 119.3, 118.9, 97.2, 58.6, 38.5, 32.0, 20.7, 18.9.

*2-((4-((4-isopropoxyphenyl)amino)-6-methylpyrimidin-2-yl)amino)propan-1-ol* (**Bc1**). Gray solid; m.p. 176–178 °C; ^1^H-NMR (500 MHz, DMSO-*d*_6_) δ 10.25 (s, 1H), 7.52 (s, 1H), 6.86 (d, *J* = 9.2 Hz, 2H), 5.97 (s, 1H), 4.57 (s, 1H), 4.53 (heptet, *J* = 6.0 Hz, 2H), 3.45 (t, *J* = 6.0 Hz, 2H), 3.36 (q, *J* = 5.8 Hz, 2H), 2.18 (s, 2H), 1.66 (quint., *J* = 6.5 Hz, 2H), 1.22 (d, *J* = 6.5 Hz, 6H); ^13^C-NMR (125 MHz, DMSO-*d*_6_) δ 161.2, 155.6, 154.6, 131.6, 123.2, 116.2, 96.2, 69.9, 58.8, 38.6, 32.3, 22.3, 19.4.

*2-((4-methyl-6-((4-morpholinophenyl)amino)pyrimidin-2-yl)amino)propan-1-ol* (**Bc2**). Gray solid; m.p. 162–166 °C; ^1^H-NMR (500 MHz, DMSO-*d*_6_) δ 9.19 (s, 1H), 7.48 (d, *J* = 8.0 Hz, 2H), 6.85 (d, *J* = 9.2 Hz, 2H), 5.77 (s, 1H), 4.54 (s, 1H), 3.69 (t, *J* = 4.6 Hz, 4H), 3.42 (t, *J* = 6.0 Hz, 2H), 3.27 (q, *J* = 6.3 Hz, 2H), 2.99 (t, *J* = 4.6 Hz, 2H), 2.06 (s, 1H), 1.63 (quint., *J* = 6.3 Hz, 6H); ^13^C-NMR (125 MHz, DMSO-*d*_6_) δ 161.2, 155.6, 154.6, 131.6, 123.2, 116.2, 96.2, 69.9, 58.8, 38.6, 32.3, 22.3, 19.4.

*2-((4-methyl-6-((4-(phenylamino)phenyl)amino)pyrimidin-2-yl)amino)propan-1-ol* (**Bc3**). Blue solid; m.p. 104–108 °C; ^1^H-NMR (500 MHz, DMSO-*d*_6_) δ 9.77 (s, 1H), 8.05 (s, 1H), 7.54 (d, *J* = 8.6 Hz, 2H), 7.15 (t, *J* = 7.7 Hz, 2H), 7.01 (t, *J* = 8.6 Hz, 2H), 6.98 (t, *J* = 8.0 Hz, 2H), 6.72 (t, *J* = 7.2 Hz, 2H), 5.90 (s, 1H), 4.59 (s, 1H), 3.44 (t, *J* = 6.0 Hz, 4H), 3.32 (q, *J* = 5.7 Hz, 2H), 2.01 (s, 3H), 1.65 (quint., *J* = 6.3 Hz, 2H); ^13^C-NMR (125 MHz, DMSO-*d*_6_) δ 172.7, 161.4, 158.7, 144.5, 139.0, 132.3, 129.6, 122.2, 119.5, 118.3, 116.3, 95.3, 59.0, 38.6, 32.7, 21.6.

*2-((4-methyl-6-((4-phenoxyphenyl)amino)pyrimidin-2-yl)amino)propan-1-ol* (**Bc4**). White solid; ^1^H-NMR (500 MHz, DMSO-*d*_6_) δ 9.10 (s, 1H), 7.71 (d, *J* = 8.6 Hz, 2H), 7.31 (td, *J* = 9.2, 2.3 Hz, 2H), 7.03 (t, *J* = 7.4 Hz, 1H), 6.93-6.91 (m, 4H), 6.63 (brs, 1H), 5.83 (s, 1H), 4.44 (s, 1H), 3.43 (t, *J* = 6.3 Hz, 2H), 3.28 (q, *J* = 6.9 Hz, 2H), 2.06 (s, 3H), 1.64 (quint., *J* = 6.4 Hz, 2H); ^13^C-NMR (125 MHz, DMSO-*d*_6_) δ 164.7, 162.2, 161.5, 158.2, 150.7, 137.3, 130.4, 123.2, 121.4, 120.0, 118.1, 95.0, 59.2, 38.5, 33.1, 23.9.

*2-((4-((4-(4-chlorophenoxy)phenyl)amino)-6-methylpyrimidin-2-yl)amino)propan-1-ol* (**Bc5**). White solid; m.p. 142–144 °C; ^1^H-NMR (500 MHz, DMSO-*d*_6_) δ 9.10 (s, 1H), 7.73 (d, *J* = 9.2 Hz, 2H), 7.34 (dt, *J* = 9.2, 2.9 Hz, 2H), 6.94 (d, *J* = 8.6 Hz, 2H), 6.93 (dt, *J* = 9.2, 2.3 Hz, 2H), 6.62 (brs, 1H), 5.82 (s, 1H), 4.41 (brs, 1H), 3.43 (t, *J* = 6.3 Hz, 2H), 3.29 (q, *J* = 6.5 Hz, 2H), 2.06 (s, 3H), 1.64 (quint., *J* = 6.3 Hz, 2H), ^13^C-NMR (125 MHz, DMSO-*d*_6_) δ 164.9, 162.2, 161.5, 157.3, 150.2, 137.7, 130.2, 126.8, 121.3, 120.2, 119.6, 94.7, 59.2, 49.1, 38.4, 33.1, 23.8. 

*3-((4-methyl-6-((4-(p-tolyloxy)phenyl)amino)pyrimidin-2-yl)amino)propan-1-ol* (**Bc6**). White solid; m.p. 136–138 °C; ^1^H-NMR (500 MHz, DMSO-*d*_6_) δ 9.03 (s, 1H), 7.68 (d, *J* = 8.6 Hz, 2H), 7.10 (d, *J* = 8.6 Hz, 2H), 6.88 (dt, *J* = 9.2, 2.6 Hz, 2H), 6.82 (dt, *J* = 8.6, 2.3 Hz, 2H), 6.59 (brs, 1H), 5.81 (s, 1H), 4.43 (s, 1H), 3.43 (t, *J* = 6.3 Hz, 2H), 3.28 (q, *J* = 6.5 Hz, 2H), 2.22 (s, 3H), 2.06 (s, 3H), 1.64 (quint., *J* = 6.4 Hz, 2H); ^13^C-NMR (125 MHz, DMSO-*d*_6_) δ 164.9, 162.3, 161.5, 155.8, 151.4, 137.0, 132.3, 130.7, 121.4, 119.5, 118.3, 94.6, 59.2, 38.5, 33.1, 24.0, 20.7.

*4-((2-((4-isopropoxyphenyl)amino)-6-methylpyrimidin-4-yl)amino)butan-1-ol* (**Ad1**). Gray solid; m.p. 170–172 °C; ^1^H-NMR (500 MHz, DMSO-*d*_6_) δ 10.19 (s, 1H), 9.04 (s, 1H), 7.41 (d, *J* = 8.6 Hz, 2H), 6.87 (d, *J* = 8.6 Hz, 2H), 5.99 (s, 1H), 4.51 (heptet, *J* = 5.9 Hz, 1H), 4.46 (s, 1H), 3.36 (t, *J* = 6.0 Hz, 2H), 3.29 (m, 2H), 2.18 (s, 3H), 1.53 (quint., *J* = 7.0 Hz, 2H), 1.41 (quint., *J* = 6.7 Hz, 2H), 1.20 (quint., *J* = 5.8 Hz, 6H); ^13^C-NMR (125 MHz, DMSO-*d*_6_) δ 163.0, 154.5, 153.1, 151.9, 130.5, 122.7, 116.4, 96.9, 69.9, 60.8, 41.1, 30.4, 25.6, 22.3, 18.9.

*4-((6-methyl-2-((4-morpholinophenyl)amino)pyrimidin-4-yl)amino)butan-1-ol* (**Ad2**). Black solid; m.p. 160–162 °C; ^1^H-NMR (500 MHz, DMSO-*d*_6_) δ 9.95 (s, 1H), 8.70 (s, 1H), 7.41 (d, *J* = 8.6 Hz, 2H), 6.89 (d, *J* = 8.1 Hz, 2H), 5.92 (s, 1H), 4.44 (s, 1H), 3.69 (brs, 4H), 3.37 (t, *J* = 6.3 Hz, 2H), 3.30 (s, 2H), 3.02 (brs, 4H), 2.17 (s, 3H), 1.53 (quint., *J* = 7.2 Hz, 2H), 1.42 (quint., *J* = 7.2 Hz, 2H); ^13^C-NMR (125 MHz, DMSO-*d*_6_) δ 163.1, 153.8, 148.1, 130.1, 122.1, 115.9, 96.7, 66.6, 49.2, 41.0, 30.4, 25.7, 19.4.

*4-((6-methyl-2-((4-(phenylamino)phenyl)amino)pyrimidin-4-yl)amino)butan-1-ol* (**Ad3**). Dark blue solid; m.p. 222–224 °C; ^1^H-NMR (500 MHz, DMSO-*d*_6_) δ 10.08 (s, 1H), 8.99 (s, 1H), 8.23 (s, 1H), 7.41 (d, *J* = 6.3 Hz, 2H), 7.15 (t, *J* = 7.2 Hz, 2H), 7.06 (d, *J* = 8.0 Hz, 2H), 7.01 (d, *J* = 7.5 Hz, 2H), 6.73 (t, *J* = 6.9 Hz, 1H), 5.99 (s, 1H), 4.50 (s, 1H), 3.38 (t, *J* = 5.8 Hz, 2H), 3.30 (brs, 2H), 2.16 (s, 3H), 1.53 (quint., *J* = 6.3 Hz, 2H), 1.43 (quint., *J* = 6.9 Hz, 2H); ^13^C-NMR (125 MHz, DMSO-*d*_6_) δ 163.0, 153.3, 152.4, 144.2, 140.2, 130.2, 129.6, 122.3, 119.7, 117.9, 116.6, 96.8, 60.8, 41.1, 30.4, 25.7, 19.2.

*4-((6-methyl-2-((4-phenoxyphenyl)amino)pyrimidin-4-yl)amino)butan-1-ol* (**Ad4**). White solid; m.p. 156–158 °C; ^1^H-NMR (500 MHz, DMSO-*d*_6_) δ 10.41 (s, 1H), 9.10 (s, 1H), 7.56 (d, *J* = 8.6 Hz, 2H), 7.34 (t, *J* = 8.0 Hz, 2H), 7.07 (d, *J* = 7.2 Hz, 2H), 7.01 (d, *J* = 8.6 Hz, 2H), 6.95 (t, *J* = 7.5 Hz, 1H), 6.02 (s, 1H), 4.43 (s, 1H), 3.35 (t, *J* = 6.6 Hz, 2H), 3.30 (q, *J* = 6.6 Hz, 2H), 2.21 (s, 3H), 1.53 (quint., *J* = 7.3 Hz, 2H), 1.41 (quint., *J* = 6.9 Hz, 2H); ^13^C-NMR (125 MHz, DMSO-*d*_6_) δ 163.0, 157.5, 153.1, 153.0, 151.9, 133.4, 130.5, 123.7, 122.8, 119.9, 118.6, 97.2, 60.8, 41.2, 30.4, 25.6, 18.9.

*4-((2-((4-(4-chlorophenoxy)phenyl)amino)-6-methylpyrimidin-4-yl)amino)butan-1-ol* (**Ad5**). Gray solid; m.p. 142–144 °C; ^1^H-NMR (500 MHz, DMSO-*d*_6_) δ 10.42 (s, 1H), 9.08 (s, 1H), 7.57 (d, *J* = 9.2 Hz, 2H), 7.37 (d, *J* = 8.6 Hz, 2H), 7.04 (d, *J* = 8.6 Hz, 2H), 6.98 (d, *J* = 8.6 Hz, 2H), 6.02 (s, 1H), 4.43 (s, 1H), 3.34 (t, *J* = 6.3 Hz, 2H), 3.32 (q, *J* = 6.3 Hz, 2H), 2.21 (s, 3H), 1.53 (quint., *J* = 7.3 Hz, 2H), 1.41 (quint., *J* = 6.9 Hz, 2H); ^13^C-NMR (125 MHz, DMSO-*d*_6_) δ 163.0, 156.6, 152.9, 152.7, 151.9, 133.8, 130.3, 127.4, 122.9, 120.2, 120.2, 97.3, 60.8, 41.2, 30.4, 25.6, 18.9.

*4-((6-methyl-2-((4-(p-tolyloxy)phenyl)amino)pyrimidin-4-yl)amino)butan-1-ol* (**Ad6**). White solid; m.p. 122–124 °C; ^1^H-NMR (500 MHz, DMSO-*d*_6_) δ 10.32 (s, 1H), 9.03 (s, 1H), 7.52 (d, *J* = 8.1 Hz, 2H), 7.14 (t, *J* = 7.5 Hz, 2H), 6.96 (d, *J* = 8.0 Hz, 2H), 6.86 (d, *J* = 7.5 Hz, 2H), 6.00 (s, 1H), 4.42 (s, 1H), 3.34-3.32 (m, 4H), 2.24 (s, 3H), 2.21 (s, 3H), 1.53 (quint., *J* = 6.9 Hz, 2H), 1.41 (m, 2H); ^13^C-NMR (125 MHz, DMSO-*d*_6_) δ 163.0, 155.0, 153.9, 152.9, 151.9, 133.0, 130.9, 123.0, 119.3, 118.9, 97.2, 60.8, 41.2, 30.3, 25.6, 20.7, 18.8.

*4-((4-((4-isopropoxyphenyl)amino)-6-methylpyrimidin-2-yl)amino)butan-1-ol* (**Bd1**). Purple solid; m.p. 153–155 °C; ^1^H-NMR (500 MHz, DMSO-*d*_6_) δ 10.74 (s, 1H), 7.92 (s, 1H), 7.58 (brs, 2H), 6.88 (d, *J* = 8.6 Hz, 2H), 6.10 (s, 1H), 4.54 (heptet, *J* = 6.1 Hz, 2H), 4.45 (s, 1H), 3.38 (t, *J* = 6.3 Hz, 2H), 3.32 (q, *J* = 6.3 Hz, 2H), 2.21 (s, 3H), 1.55 (quint., *J* = 7.3 Hz, 2H), 1.43 (quint., *J* = 7.0 Hz, 2H), 1.21 (d, *J* = 5.7 Hz, 6H); ^13^C-NMR (125 MHz, DMSO-*d*_6_) *δ* 161.0, 154.8, 152.6, 131.3, 127.1, 123.3, 116.2, 96.6, 69.9, 60.8, 41.2, 30.2, 25.9, 22.3, 18.9.

*4-((4-methyl-6-((4-morpholinophenyl)amino)pyrimidin-2-yl)amino)butan-1-ol* (**Bd2**). Pink solid; m.p. 208–210 °C; ^1^H-NMR (500 MHz, DMSO-*d*_6_) δ 10.77 (s, 1H), 7.94 (s, 1H), 7.57 (brs, 2H), 6.90 (d, *J* = 9.2 Hz, 2H), 6.11 (s, 1H), 4.47 (s, 1H), 3.68 (t, *J* = 4.6 Hz, 4H), 3.37 (t, *J* = 6.3 Hz, 2H), 3.32 (q, *J* = 6.1 Hz, 2H), 3.03 (t, *J* = 4.3 Hz, 2H), 2.20 (s, 3H), 1.53 (quint., *J* = 7.2 Hz, 2H), 1.43 (quint., *J* = 6.9 Hz, 2H); ^13^C-NMR (125 MHz, DMSO-*d*_6_) δ 160.7, 154.9, 152.2, 148.5, 130.5, 122.5, 115.6, 96.7, 66.5, 60.5, 49.0, 41.1, 30.2, 25.9, 18.9.

*4-((4-methyl-6-((4-(phenylamino)phenyl)amino)pyrimidin-2-yl)amino)butan-1-ol* (**Bd3**). Dark blue solid; m.p. 192–194 °C; ^1^H-NMR (500 MHz, DMSO-*d*_6_) δ 10.77 (s, 1H), 8.25 (s, 1H), 7.93 (brs, 2H), 7.17 (t, *J* = 7.4 Hz, 2H), 7.05 (d, *J* = 8.6 Hz, 2H), 7.02 (d, *J* = 8.0 Hz, 2H), 6.76 (t, *J* = 7.2 Hz, 1H), 6.13 (s, 1H), 4.47 (s, 1H), 3.38 (t, *J* = 6.0 Hz, 2H), 3.32 (q, *J* = 5.8 Hz, 2H), 2.21 (s, 3H), 1.55 (quint., *J* = 7.0 Hz, 2H), 1.44 (quint., *J* = 6.7 Hz, 2H); ^13^C-NMR (125 MHz, DMSO-*d*_6_) δ 160.7, 154.8, 152.2, 143.9, 140.6, 130.8, 129.6, 122.8, 120.0, 117.4, 116.9, 96.8, 60.8, 41.2, 30.3, 25.9, 18.8.

*4-((4-methyl-6-((4-phenoxyphenyl)amino)pyrimidin-2-yl)amino)butan-1-ol* (**Bd4**). White solid; m.p. 140–142 °C; ^1^H-NMR (500 MHz, DMSO-*d*_6_) δ 9.12 (s, 1H), 7.70 (d, *J* = 9.2 Hz, 2H), 7.31 (t, *J* = 8.0 Hz, 2H), 7.04 (t, *J* = 7.4 Hz, 1H), 6.93 (d, *J* = 8.6 Hz, 2H), 6.92 (d, *J* = 8.0 Hz, 2H), 6.71 (brs, 1H), 5.82 (s, 1H), 4.36 (brs, 1H), 3.37 (t, *J* = 6.6 Hz, 2H), 3.21 (q, *J* = 6.7 Hz, 2H), 2.06 (s, 1H), 1.51 (quint., *J* = 7.2 Hz, 2H), 1.42 (quint., *J* = 7.0 Hz, 2H); ^13^C-NMR (125 MHz, DMSO-*d*_6_) δ 164.5, 161.9, 161.5, 158.2 150.7, 137.3, 130.4, 123.2, 121.4, 120.0, 118.0, 94.7, 61.1, 41.2, 30.6, 26.5, 23.8.

*4-((4-((4-(4-chlorophenoxy)phenyl)amino)-6-methylpyrimidin-2-yl)amino)butan-1-ol* (**Bd5**). White solid; m.p. 164–166 °C; ^1^H-NMR (500 MHz, DMSO-*d*_6_) δ 10.41 (s, 1H), 9.10 (s, 1H), 7.56 (d, *J* = 8.6 Hz, 2H), 7.34 (t, *J* = 8.0 Hz, 2H), 7.07 (d, *J* = 7.2 Hz, 2H), 7.01 (d, *J* = 8.6 Hz, 2H), 6.95 (t, *J* = 7.5 Hz, 1H), 6.02 (s, 1H), 4.43 (s, 1H), 3.35 (t, *J* = 6.6 Hz, 2H), 3.30 (q, *J* = 6.6 Hz, 2H), 2.21 (s, 3H), 1.53 (quint., *J* = 7.3 Hz, 2H), 1.41 (quint., *J* = 6.9 Hz, 2H); ^13^C-NMR (125 MHz, DMSO-*d*_6_) δ 163.0, 157.5, 153.1, 153.0, 151.9, 133.4, 130.5, 123.7, 122.8, 119.9, 118.6, 97.2, 60.8, 41.2, 30.4, 25.6, 18.9.

*4-((4-methyl-6-((4-(p-tolyloxy)phenyl)amino)pyrimidin-2-yl)amino)butan-1-ol* (**Bd6**). White solid; ^1^H-NMR (500 MHz, DMSO-*d*_6_) δ 9.22 (s, 1H), 7.69 (d, *J* = 9.2 Hz, 2H), 7.10 (d, *J* = 8.0 Hz, 2H), 6.89 (d, *J* = 9.2 Hz, 2H), 6.82 (d, *J* = 8.6 Hz, 2H), 6.74 (s, 1H), 5.84 (s, 1H), 4.34 (brs, 1H), 3.38 (t, *J* = 6.3 Hz, 2H), 3.23 (q, *J* = 6.7 Hz, 2H), 2.21 (s, 3H), 2.07 (s, 3H), 1.52 (quint., *J* = 7.2 Hz, 2H), 1.43 (quint., *J* = 7.0 Hz, 2H); ^13^C-NMR (125 MHz, DMSO-*d*_6_) δ 163.8, 161.5, 155.7, 151.5, 136.8, 132.3, 130.7, 121.5, 119.5, 118.3, 94.7, 61.2, 41.2, 30.6, 26.5, 23.2, 20.7.

*5-((2-((4-isopropoxyphenyl)amino)-6-methylpyrimidin-4-yl)amino)pentan-1-oll* (**Ae1**). Gray solid; ^1^H-NMR (500 MHz, DMSO-*d*_6_) δ 8.71 (s, 1H), 7.61 (dt, *J* = 8.6, 3.5 Hz, 2H), 7.03 (s, 1H), 6.74 (dt, *J* = 9.2, 3.5 Hz, 2H), 5.70 (s, 1H), 4.44 (heptet, *J* = 6.0 Hz, 1H), 4.34 (s, 1H), 3.35–3.32 (m, 4H), 2.05 (s, 3H), 1.49 (quint., *J* = 7.3 Hz, 2H), 1.41 (quint., *J* = 7.0 Hz, 2H), 1.31 (quint., *J* = 7.2 Hz, 2H), 1.19 (d, *J* = 6.3 Hz, 2H); ^13^C-NMR (125 MHz, DMSO-*d*_6_) δ 163.6, 159.7, 152.0, 135.1, 120.4, 116.2, 69.9, 61.2, 40.5, 32.8, 29.5, 23.7, 22.4.

*5-((6-methyl-2-((4-morpholinophenyl)amino)pyrimidin-4-yl)amino)pentan-1-ol* (**Ae2**). Gray solid; m.p. 148–150 °C; ^1^H-NMR (500 MHz, DMSO-*d*_6_) δ 8.71 (s, 1H), 7.58 (d, *J* = 8.6 Hz, 2H), 7.09 (s, 1H), 6.80 (d, *J* = 9.2 Hz, 2H), 5.70 (s, 1H), 4.34 (brs, 1H), 3.68 (t, *J* = 4.6 Hz, 4H), 3.35 (brs, 2H), 3.23 (brs, 2H), 2.96 (t, *J* = 4.6 Hz, 4H), 2.06 (s, 3H), 1.49 (quint., *J* = 7.2 Hz, 2H), 1.41 (quint., *J* = 7.0 Hz, 2H), 1.30 (quint., *J* = 7.3 Hz, 2H); ^13^C-NMR (125 MHz, DMSO-*d*_6_) δ 163.6, 159.5, 146.0, 134.3, 120.2, 116.1, 95.8, 66.7, 61.2, 49.9, 40.3, 32.8, 29.5, 23.7, 23.4.

*5-((6-methyl-2-((4-(phenylamino)phenyl)amino)pyrimidin-4-yl)amino)pentan-1-ol* (**Ae3**). Dark gray solid; m.p. 238–240 °C; ^1^H-NMR (500 MHz, DMSO-*d*_6_) δ 9.32 (s, 1H), 8.02 (s, 2H), 7.53 (s, 2H), 7.13 (s, 2H), 6.97-6.95 (m, 4H), 6.68 (s, 1H), 5.83 (s, 1H), 4.39 (brs, 1H), 3.34–3.25 (m 4H), 2.11 (s, 3H), 1.50 (s, 2H), 1.30 (2H), 1.19 (s, 2H); ^13^C-NMR (125 MHz, DMSO-*d*_6_) δ 163.4, 157.4, 145.0, 137.6, 133.5, 129.5, 124.7, 120.7, 119.0, 118.8, 115.8, 96.0, 61.1, 40.9, 32.7, 29.2, 23.6, 21.6.

*5-((6-methyl-2-((4-phenoxyphenyl)amino)pyrimidin-4-yl)amino)pentan-1-ol* (**Ae4**). White solid; ^1^H-NMR (500 MHz, DMSO-*d*_6_) δ 8.94 (s, 1H), 7.79 (dt, *J* = 9.2, 2.6 Hz, 2H), 7.29 (t, *J* = 8.0 Hz, 2H), 7.04 (brs, 1H), 7.00 (t, *J* = 7.2 Hz, 1H), 6.90-6.88 (m, 4H), 5.75 (s, 1H), 4.34 (brs, 1H), 3.35 (t, *J* = 6.3 Hz, 2H), 3.24 (brs, 2H), 2.08 (s, 3H), 1.50 (quint., *J* = 7.5 Hz, 2H), 1.40 (quint., *J* = 7.0 Hz, 2H), 1.31 (quint., *J* = 7.3 Hz, 2H); ^13^C-NMR (125 MHz, DMSO-*d*_6_) δ 163.7, 159.9, 158.6, 149.6, 138.3, 130.3, 122.9, 120.2, 120.0, 117.7, 96.3, 61.2, 49.0, 40.7, 32.8, 29.4, 23.7, 23.7.

*5-((2-((4-(4-chlorophenoxy)phenyl)amino)-6-methylpyrimidin-4-yl)amino)pentan-1-ol* (**Ae5**). Gray solid; m.p. 146–148 °C; ^1^H-NMR (500 MHz, DMSO-*d*_6_) δ 8.94 (s, 1H), 7.81 (d, *J* = 9.2 Hz, 2H), 7.32 (dt, *J* = 8.6, 2.6 Hz, 2H), 6.98 (s, 1H), 6.90 (d, *J* = 9.2 Hz, 2H), 6.90 (d, *J* = 8.6 Hz, 2H), 5.74 (s, 1H), 4.34 (brs, 1H), 4.35 (t, *J* = 6.3 Hz, 2H), 3.24 (brs, 2H), 2.07 (s, 3H), 1.50 (quint., *J* = 7.3 Hz, 2H), 1.40 (quint., *J* = 6.9 Hz, 2H), 1.31 (quint., *J* = 7.3 Hz, 2H); ^13^C-NMR (125 MHz, DMSO-*d*_6_) δ 163.7, 160.0, 157.6, 149.1, 138.8, 130.1, 126.5, 120.2, 120.1, 119.2, 96.0, 61.2, 49.0, 40.7, 32.8, 29.5, 23.8, 23.7.

*5-((6-methyl-2-((4-(p-tolyloxy)phenyl)amino)pyrimidin-4-yl)amino)pentan-1-ol* (**Ae6**). White solid; ^1^H-NMR (500 MHz, DMSO-*d*_6_) δ 8.90 (s, 1H), 7.78 (dt, *J* = 9.2, 2.6 Hz, 2H), 7.08 (d, *J* = 8.0 Hz, 2H), 7.00 (s, 1H), 6.85 (dt, *J* = 9.2, 2.6 Hz, 2H), 6.79 (dt, *J* = 8.6, 2.6 Hz, 2H), 5.74 (s, 1H), 4.36 (brs, 1H), 3.36 (t, *J* = 6.3 Hz, 2H), 3.27 (brs, 2H), 2.20 (s, 3H), 2.07 (s, 3H), 1.50 (quint., *J* = 7.3 Hz, 2H), 1.41 (quint., *J* = 6.9 Hz, 2H), 1.31 (quint., *J* = 7.3 Hz, 2H); ^13^C-NMR (125 MHz, DMSO-*d*_6_) δ 163.7, 159.9, 156.2, 150.3, 138.0, 131.9, 130.6, 120.2, 119.5, 117.9, 96.3, 61.2, 49.1, 40.8, 32.8, 29.5, 23.7, 23.7, 20.6.

*5-((4-((4-isopropoxyphenyl)amino)-6-methylpyrimidin-2-yl)amino)pentan-1-ol* (**Be1**). Black solid; m.p. 138–140 °C; ^1^H-NMR (500 MHz, DMSO-*d*_6_) δ 8.67 (s, 1H), 7.62 (dt, *J* = 8.6, 2.6 Hz, 2H), 6.96 (s, 1H), 6.73 (dt, *J* = 9.2, 2.6 Hz, 2H), 5.70 (s, 1H), 4.43 (heptet, *J* = 5.0 Hz, 1H), 4.35 (t, *J* = 6.6 Hz, 2H), 3.23 (brs, 2H), 2.05 (s, 1H), 1.49 (quint., *J* = 7.3 Hz, 2H), 1.42 (quint., *J* = 7.0 Hz, 2H), 1.31 (quint., *J* = 7.6 Hz, 2H), 1.18 (d, *J* = 5.8 Hz, 2H); ^13^C-NMR (125 MHz, DMSO-*d*_6_) δ 163.7, 160.0, 151.9, 135.2, 120.3, 116.2, 95.7, 69.9, 61.2, 49.1, 32.8, 29.5, 23.7, 22.4.

*5-((4-methyl-6-((4-morpholinophenyl)amino)pyrimidin-2-yl)amino)pentan-1-ol* (**Be2**). White solid; ^1^H-NMR (500 MHz, DMSO-*d*_6_) δ 9.07 (s, 1H), 7.49 (d, *J* = 8.6 Hz, 2H), 6.84 (d, *J* = 9.2 Hz, 2H), 6.74 (s, 1H), 5.77 (s, 1H), 4.35 (brs, 1H), 3.68 (t, *J* = 4.6 Hz, 4H), 3.35 (t, *J* = 6.3 Hz, 2H), 3.20 (q, *J* = 6.9 Hz, 2H), 2.99 (t, *J* = 4.6 Hz, 2H), 2.05 (s, 3H), 1.48 (quint., *J* = 7.3 Hz, 2H), 1.41 (quint., *J* = 7.0 Hz, 2H), 1.29 (quint., *J* = 7.5 Hz, 2H); ^13^C-NMR (125 MHz, DMSO-*d*_6_) δ 161.6, 146.9, 142.9, 142.7, 121.5, 118.0, 116.0, 115.3, 66.8, 66.6, 61.2, 51.1, 49.6, 49.1, 41.3, 32.9, 29.7, 23.6.

*2-((4-methyl-6-((4-(phenylamino)phenyl)amino)pyrimidin-2-yl)amino)propan-1-ol* (**Be3**). White solid; ^1^H-NMR (500 MHz, DMSO-*d*_6_) δ 8.73 (s, 1H), 7.80 (s, 1H), 7.62 (d, *J* = 9.2 Hz, 2H), 7.11 (t, *J* = 7.7 Hz, 2H), 6.99 (brs, 1H), 6.93 (dt, *J* = 8.6, 2.6 Hz, 2H), 6.90 (dd, *J* = 8.6, 1.2 Hz, 2H), 6.66 (t, *J* = 7.5 Hz, 1H), 5.70 (s, 1H), 4.33 (t, *J* = 5.2 Hz, 1H), 3.35 (q, *J* = 5.9 Hz, 2H), 3.23 (brs, 2H), 2.06 (s, 3H), 1.50 (quint., *J* = 7.5 Hz, 2H), 1.41 (quint., *J* = 6.9 Hz, 2H), 1.30 (quint., *J* = 7.6 Hz, 2H); ^13^C-NMR (125 MHz, DMSO-*d*_6_) δ 163.6, 145.5, 136.5, 135.6, 129.5, 120.0, 119.4, 118.6, 115.3, 104.0, 61.2, 32.8, 29.5, 23.7.

*5-((4-methyl-6-((4-phenoxyphenyl)amino)pyrimidin-2-yl)amino)pentan-1-ol* (**Be4**). White solid; ^1^H-NMR (500 MHz, DMSO-*d*_6_) δ 9.16 (s, 1H), 7.71 (d, *J* = 9.2 Hz, 2H), 7.30 (t, *J* = 8.0 Hz, 2H), 7.04 (t, *J* = 7.2 Hz, 2H), 6.92–6.91 (m, 4H), 6.75 (brs, 1H), 5.82 (s, 1H), 4.32 (s, 1H), 3.33 (t, *J* = 6.3 Hz, 2H), 3.20 (q, *J* = 6.9 Hz, 2H), 2.07 (s, 3H), 1.48 (quint., *J* = 7.3 Hz, 2H), 1.39 (quint., *J* = 6.9 Hz, 2H), 1.28 (quint., *J* = 7.5 Hz, 2H); ^13^C-NMR (125 MHz, DMSO-*d*_6_) δ 164.3, 161.9, 161.5, 158.2, 150.8, 137.2, 130.4, 123.2, 121.4, 120.0, 118.1, 94.7, 61.2, 49.1, 41.3, 32.8, 29.6, 23.6.

*5-((4-((4-(4-chlorophenoxy)phenyl)amino)-6-methylpyrimidin-2-yl)amino)pentan-1-ol* (**Be5**). White solid; m.p. 136–138 °C; ^1^H-NMR (500 MHz, DMSO-*d*_6_) δ 9.16 (s, 1H), 7.73 (d, *J* = 9.2 Hz, 2H), 7.33 (dt, *J* = 9.2, 2.9 Hz, 2H), 6.95–6.90 (m, 4H), 6.75 (s, 1H), 5.83 (s, 1H), 4.28 (s, 1H), 3.34 (t, *J* = 6.3 Hz, 2H), 3.21 (q, *J* = 6.7 Hz, 2H), 2.07 (s, 3H), 1.49 (quint., *J* = 7.3 Hz, 2H), 1.39 (quint., *J* = 6.9 Hz, 2H), 1.29 (quint., *J* = 7.3 Hz, 2H); ^13^C-NMR (125 MHz, DMSO-*d*_6_) δ 164.6, 162.0, 161.5, 157.2, 150.3, 137.7, 130.2, 126.9, 121.3, 120.2, 119.6, 94.9, 61.2, 41.3, 32.9, 29.6, 23.6.

*5-((4-methyl-6-((4-(p-tolyloxy)phenyl)amino)pyrimidin-2-yl)amino)pentan-1-ol* (**Be6**). White solid; ^1^H-NMR (500 MHz, DMSO-*d*_6_) δ 9.44 (s, 1H), 7.68 (d, *J* = 9.2 Hz, 2H), 7.11 (d, *J* = 8.6 Hz, 2H), 6.92 (s, 1H), 6.88 (d, *J* = 8.6 Hz, 2H), 6.83 (d, *J* = 8.6 Hz, 2H), 5.86 (s, 1H), 4.32 (brs, 1H), 3.33 (t, *J* = 6.3 Hz, 2H), 3.21 (q, *J* = 6.7 Hz, 2H), 2.22 (s, 3H), 2.08 (s, 3H), 1.48 (quint., *J* = 7.5 Hz, 2H), 1.39 (quint., *J* = 7.1 Hz, 2H), 1.28 (quint., *J* = 7.8 Hz, 2H); ^13^C-NMR (125 MHz, DMSO-*d*_6_) δ 161.5, 155.6, 151.9, 136.7, 132.4, 130.7, 121.7, 119.3, 118.5, 95.1, 61.2, 41.3, 32.8, 29.5, 23.6, 22.5, 20.7.

### 3.4. Cell Culture

The A549 cells, human lung adenocarcinoma, and the NCI-H460 cells, human large-cell lung carcinoma, were obtained from American Type Culture Collection (ATCC, Manassas, VA, USA). The Fisher rat thyroid (FRT) cells stably expressing YFP-H148Q/I152L/F46L and ANO1 (friendly gifted from Prof. Wan Namkung, Yonsei University, Incheon, Korea) were cultured in Dulbecco Modified Eagle Medium (DMEM) and Ham’s F12 medium (1:1 ratio) medium supplemented with 10% fetal bovine serum (FBS), 100 U/mL penicillin, 100 µg/mL streptomycin, 0.25mg/mL G418 and 0.1 mg/mL hygromycin B. A549 and NCI-H460 were cultured in RPMI1640 medium containing 10% FBS, 100 U/mL penicillin and 100 µg/mL streptomycin. Cells were cultured at 37 °C in a humidified atmosphere of 5% CO_2_.

### 3.5. Halide Sensitive YFP Imaging

The Fisher rat thyroid (FRT) cells stably expressing YFP-H148Q/I152L/F46L and ANO1 were plated in 96-well microplates at a density of 20,000 cells per well in DMEM/F12 medium supplemented with 10% FBS, 100 U/mL penicillin and 100 µg/mL streptomycin for 24 h. The cells were washed twice with 100 µL of 1× PBS and then test compounds were applied in 100 µl NaCl solution for 20 min. Additionally, all of the compounds tested in this study were prepared using dimethyl sulfoxide (DMSO), which is widely used as a solvent in organic synthesis. After incubation, to stimulate ANO1-mediated I^−^ influx, cells were injected with 100 µL NaI solution including with 200 µM ATP at 1 s, and the fluorescence was measured once every 0.2 s and continuously measured for 6 s. The inhibitory effect of the test compound was measured by reduction of YFP fluorescence by I^−^ influx. Assays were done using SpectraMax i3x multi-microplate reader (Molecular Devices, San Jose, CA, USA) equipped with 488 nm excitation and 520 nm emission filters.

### 3.6. Cell Proliferation Assay

A549 and NCI-H460 cells were plated in 96-well microplates at a density of 7000 cells per well for 24 h. Each well was treated with test compounds and incubated at 37 °C with 5% CO_2_ for 48 h. Proliferation rates were assessed by a reagent WST-1 (Roche, Mannheim, Germany). Briefly, after removing the cell culture medium, WST-1 solution was added to the 96-well plate and re-incubated for 1 h. The soluble formazan produced by cellular reduction of WST-1 was quantified by measuring the absorbance at 490 and 690 nm (background) with SpectraMax i3x multi-microplate reader (Molecular Devices, San Jose, CA, USA).

### 3.7. Cell Viability Assay

A549 and NCI-H460 cells were plated in 24-well microplates at a density of 10,000 cells per well for 24 h. Each well was treated with test compounds and incubated at 37 °C with 5% CO_2_ for 48 h. The cells were fixed and then stained by crystal violet.

### 3.8. Western Blot Analysis

The compound-treated NCI-H460 cells were cultured on 6-well plates were rinsed twice with PBS and harvested using a PRO-PREP™ lysis buffer (iNtRON, Seoungnam, Korea) that was supplemented with phosphatase inhibitor cocktail (Thermo Scientific, Rockford, IL, USA). The proteins were separated by sodium dodecyl sulfate polyacrylamide gel electrophoresis (SDS-PAGE) on 8% gel and then transferred onto polyvinylidene fluoride (PVDF) membranes. The membranes were blocked with 5% skim milk in TBST for 1 h at room temperature. After that, the membranes were incubated with primary antibodies with 1% bovine serum albumin (BSA) in TBST at 4 °C overnight. The following primary antibodies were used: rabbit anti-ANO1 (1:1000, abcam) and rabbit anti-GAPDH (1:5000, sc-25778). Then, the membranes were washed 3 times for 10 min each and incubated with secondary antibodies (1:5000, GeneTex) for 1 h at room temperature. The membrane was then washed three times with TBST for 10 min and then visualized using the enhanced chemiluminescent (ECL) detection on an ImageQuantTM LAS-4000 imager (GE Healthcare Bio-Sciences AB, Uppsala, Sweden).

## 4. Conclusions

In summary, we designed and synthesized a series of novel ANO1 channel blockers with pyrimidine cores using a two-step combinatorial approach in a simplified manner. HTS of a focused in-house library using the halide-sensitive YFP imaging technique enabled us to discover the compound **Aa3**, which was shown to be a dose-dependent ANO1 channel blocker. The anti-cancer activity of **Aa3** was also confirmed in ANO1 overexpressing NCI-H460 cells. This study could contribute to the development of treatments for various cancers and diseases, including lung cancer. 

## Figures and Tables

**Table 1:** Chemical structures of synthesized compounds.

A or B	R^1^	R^2^
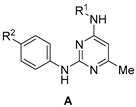	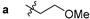	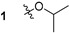
	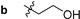	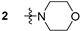
	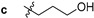	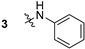
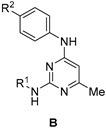	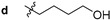	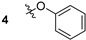
	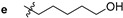	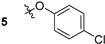
		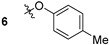

## Supplementary Materials

The following are available online, Spectral data of ^1^H-NMR and ^13^C-NMR.
