# Supplementary material for: Design of Anticancer 2,4-Diaminopyrimidines as Novel Anoctamin 1 (ANO1) Ion Channel Blockers
Source: Molecules. 2020 Nov 6;25(21):5180. doi: 10.3390/molecules25215180 (PMC7664333; doi:10.3390/molecules25215180)
Supplement: Supplementary file 1 [file molecules-25-05180-s001.pdf]

Supporting Information

Design of Anticancer 2,4-Diaminopyrimidines as Novel Anoctamin 1 (ANO1) Ion Channel Blockers

Taewoo Kim [1,†], Sinyoung Cho [1,†], Haejun Oh [1,†], Joonseong Hur [2], Haedong Kim [1], Young-Ho Choi [1], Seongho Jeon [1], Young Duk Yang [1,*] and Seok-Ho Kim [1,*]

[1] Department of Pharmacy, College of Pharmacy and Institute of Pharmaceutical Sciences, CHA University, 120 Haeryong-ro, Pocheon, Gyeonggi-do 11160, Korea; taewookim@snu.ac.kr (T.K.); jsy7122@naver.com (S.C.); dhgowns2@naver.com (H.O.); eanby12@nate.com (H.K.); dudgh705@gmail.com (Y.-H.C.); cmb_jsh@naver.com (S.J.)

[2] Natural Products Research Institute, Korea Institute of Science and Technology (KIST), 679 Saimdang-ro, Gangneung 25451, Korea; hjs1120@kist.re.kr

* Correspondence: ntsky0816@ cha.ac.kr (Y.D.Y.); ksh3410@cha.ac.kr (S.-H.K.); Tel.: +82-31-881-7170 (Y.D.Y.); +82-31-881-7169 (S.-H.K.)

† These authors contributed equally to this work.

Table of Contents

Copies of ^{1}H-NMR and ^{13}C-NMR Spectra -- S2-S15

Cytotoxic activity for **Bd5**, **Ae5** and **Ae6** in NCI-H460 cells -- S16

The **Aa3** compound inhibits the A23187-induced ANO1 activity --- S17

The expression levels of endogenous ANO1 protein between A549 and NCI-H460 cells--------------S18

^{1}H-NMR and ^{13}C-NMR spectra

4-Chloro-*N*-(2-methoxyethyl)-6-methylpyrimidin-2-amine (**Aa**)

^{1}H-NMR ($CDCl_3$, 500 MHz)

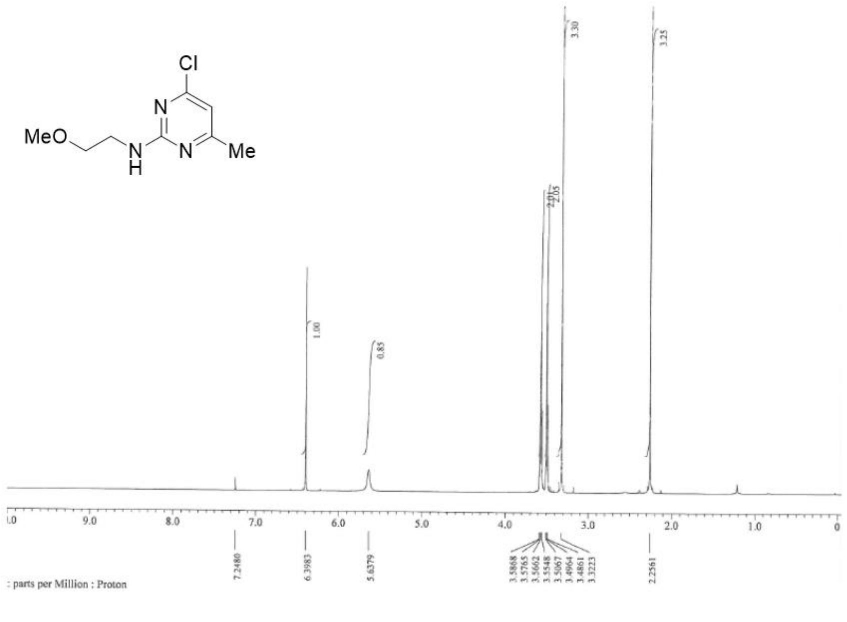

¹³C-NMR (CDCl₃, 125 MHz)

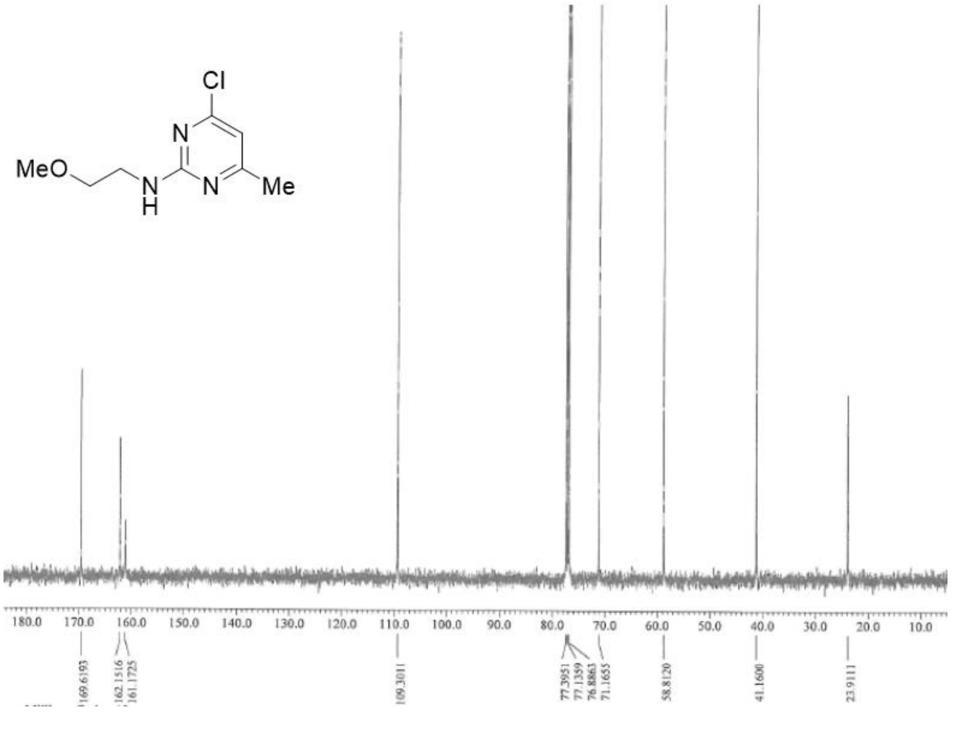

2-Chloro-*N*-(2-methoxyethyl)-6-methylpyrimidin-4-amine (**Ba**)

^{1}H-NMR ($CDCl_3$, 500 MHz)
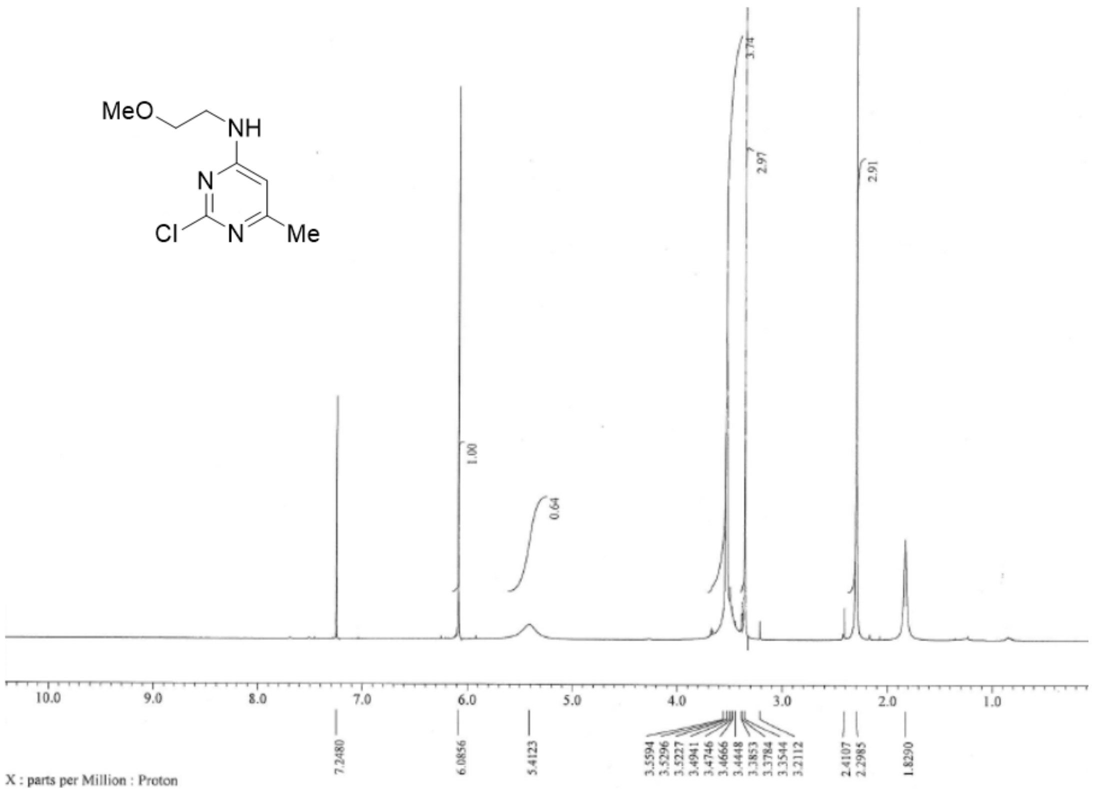
^{13}C-NMR ($CDCl_3$, 125 MHz)

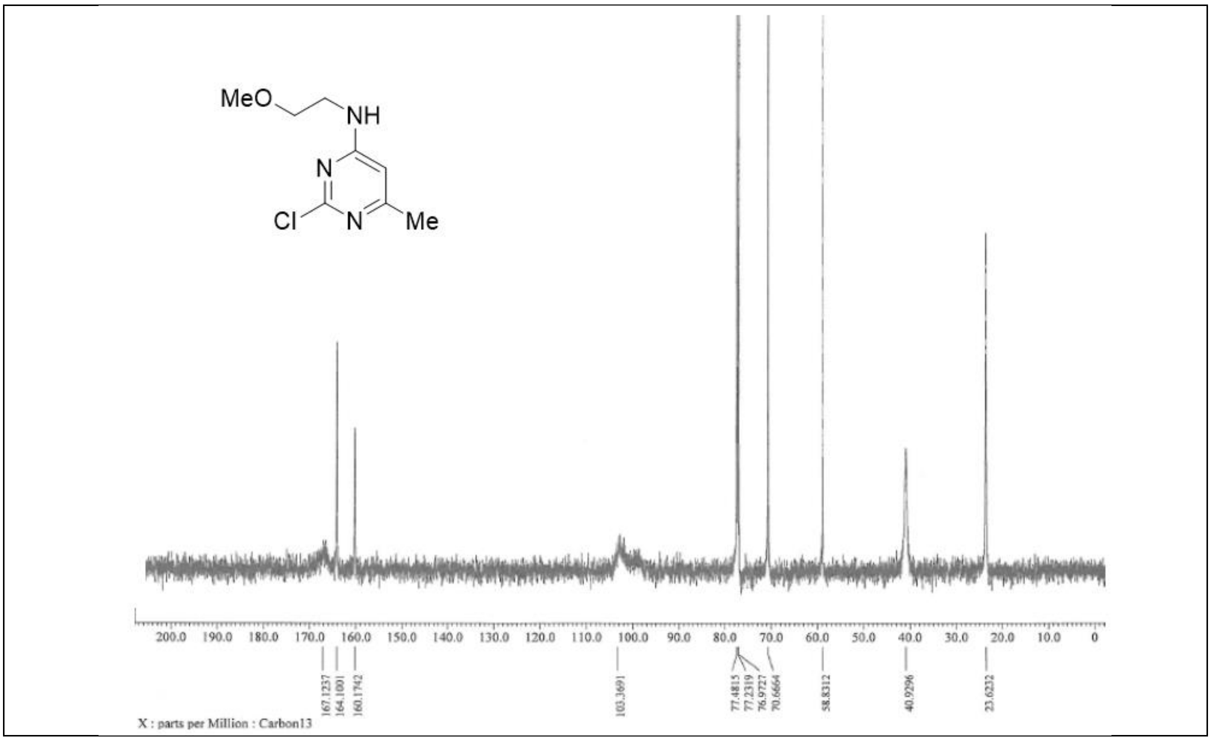

N[2]-(4-isopropoxyphenyl)-*N*[4]-(2-methoxyethyl)-6-methylpyrimidine-2,4-diamine (**Aa1**)

^{1}H-NMR (DMSO-d$_6$, 500 MHz)

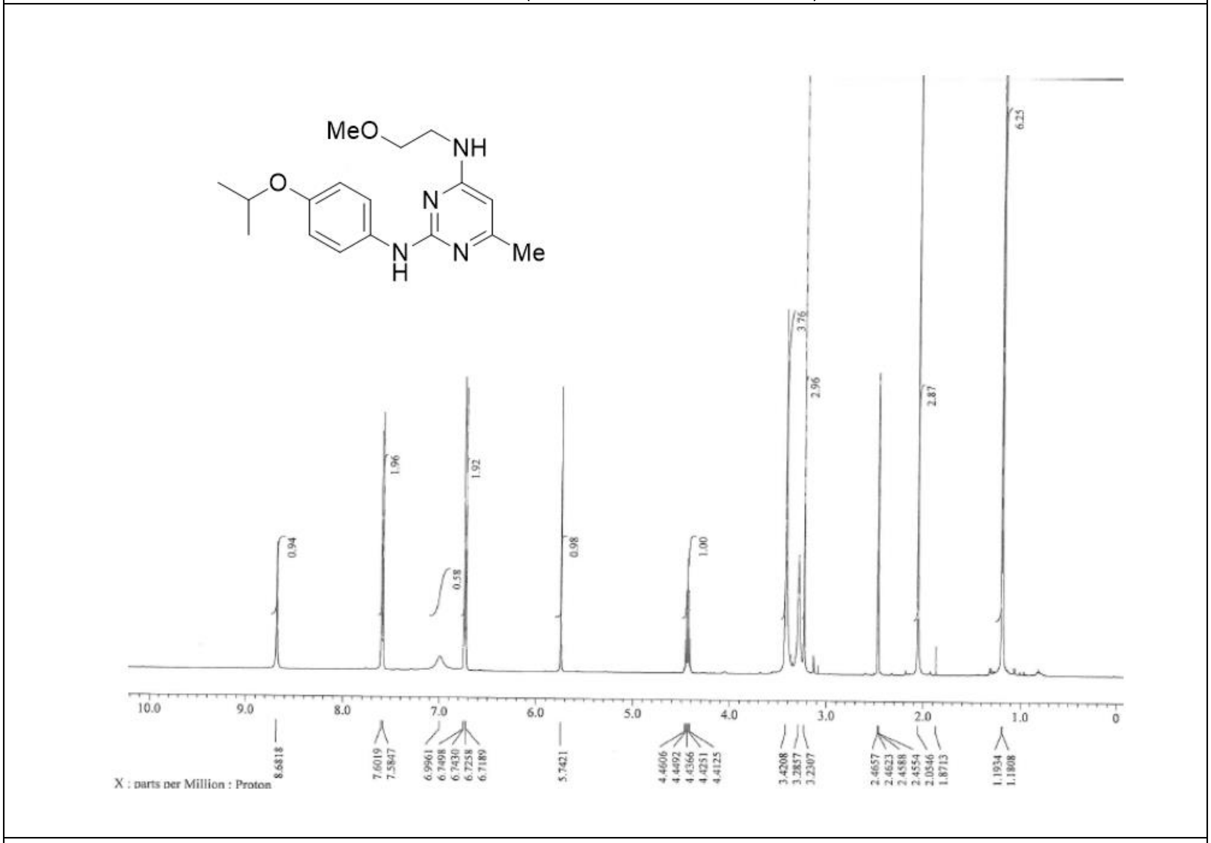

^{13}C-NMR (DMSO-d$_6$, 125 MHz)

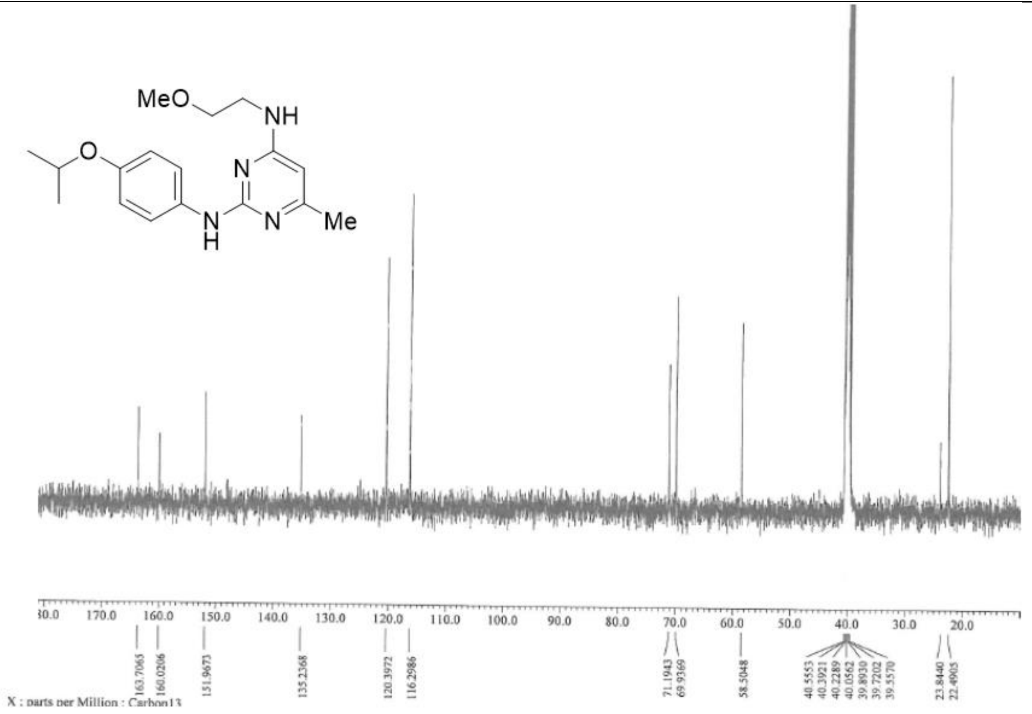

N^4-(2-methoxyethyl)-6-methyl-N^2-(4-morpholinophenyl)pyrimidine-2,4-diamine (**Aa2**)

^{1}H-NMR (DMSO-d$_6$, 500 MHz)
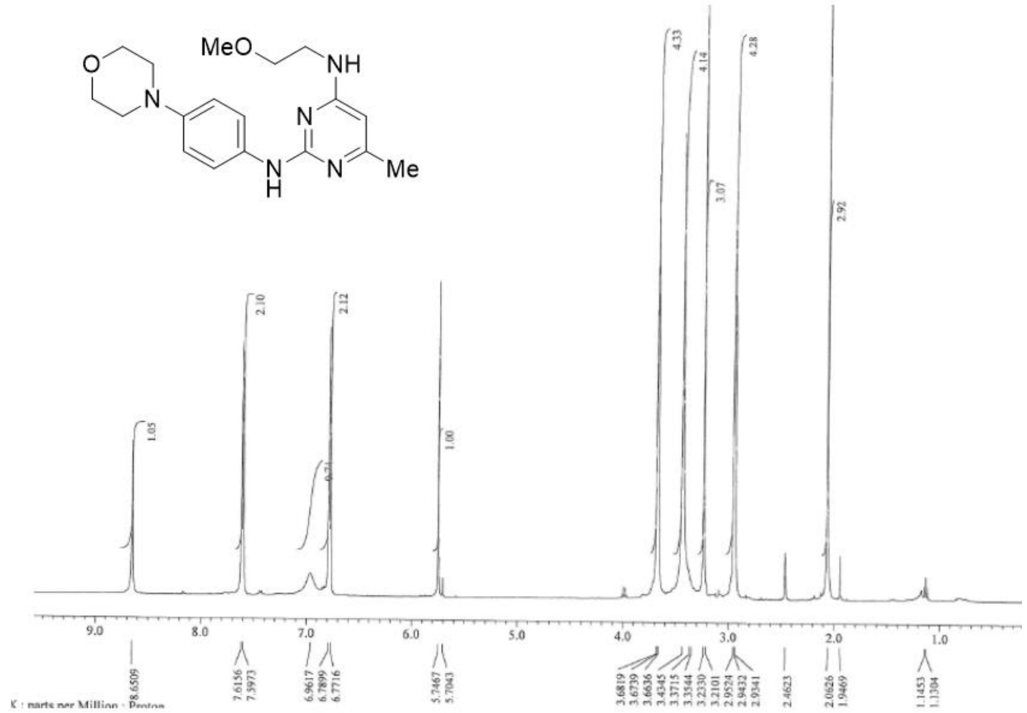
^{13}C-NMR (DMSO-d$_6$, 125 MHz)

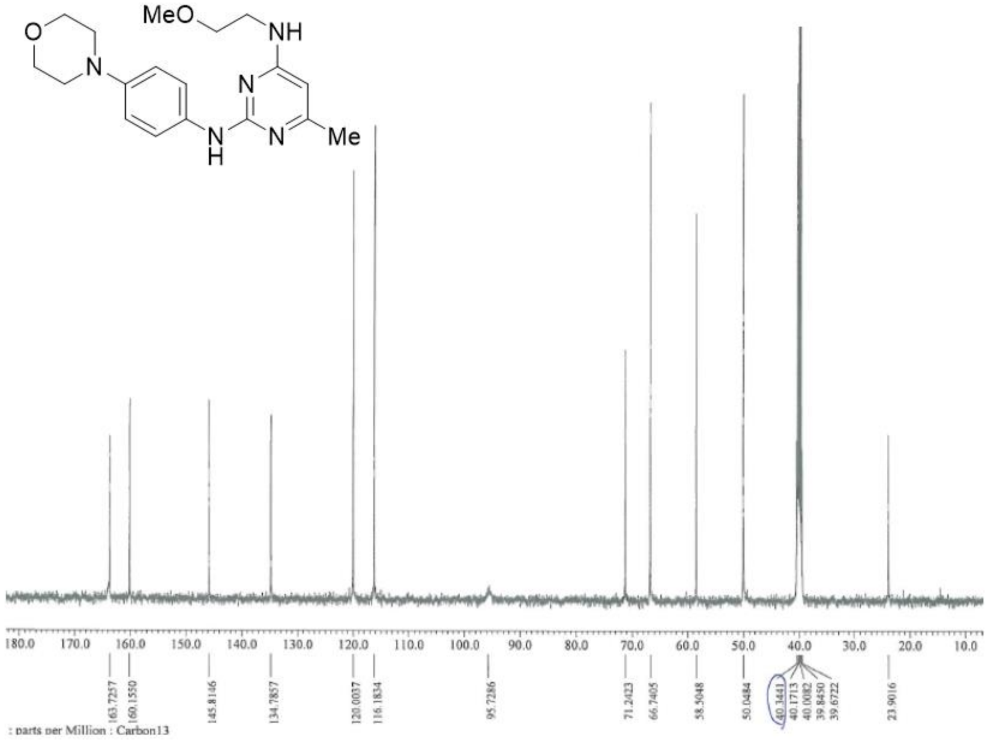

N^4-(2-methoxyethyl)-6-methyl-N^2-(4-(phenylamino)phenyl)pyrimidine-2,4-diamine (**Aa3**).

^{1}H-NMR ($CDCl_3$, 500 MHz)

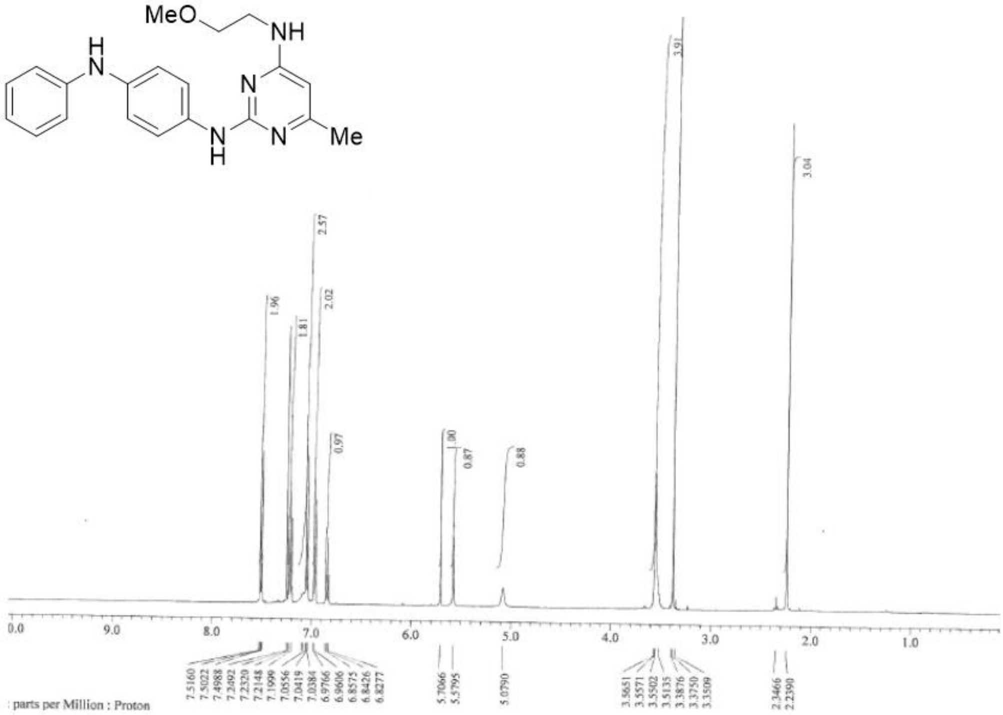

^{13}C-NMR ($CDCl_3$, 125 MHz)

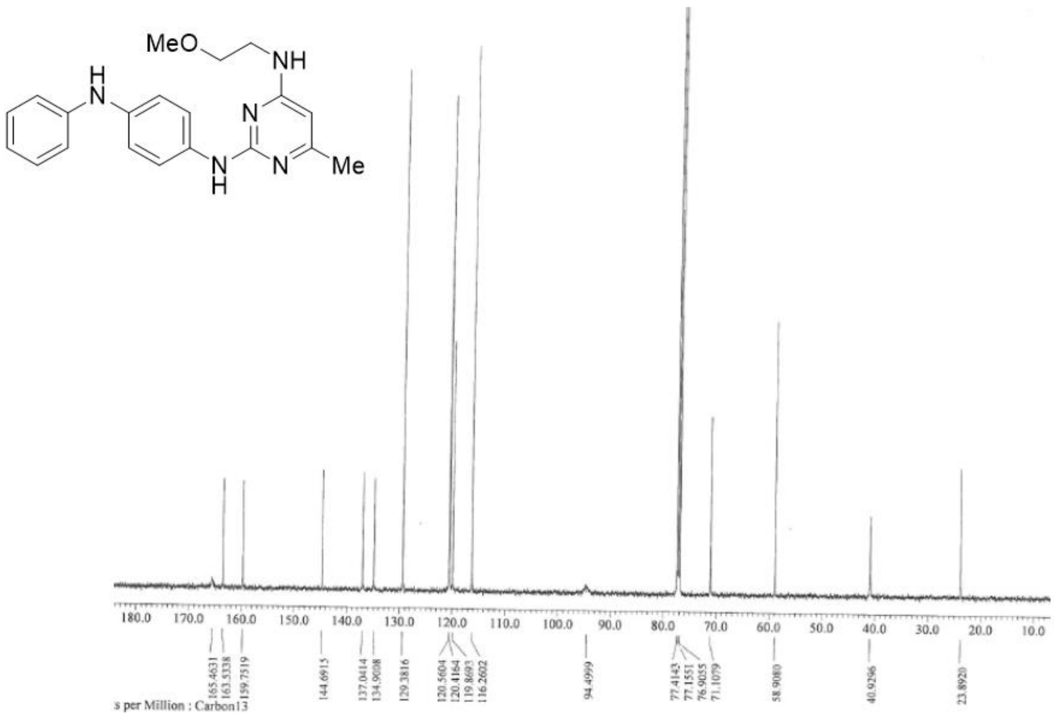

N[4]-(2-methoxyethyl)-6-methyl-*N*[2]-(4-phenoxyphenyl)pyrimidine-2,4-diamine (**Aa4**).

^{1}H-NMR (DMSO-d_6, 500 MHz)

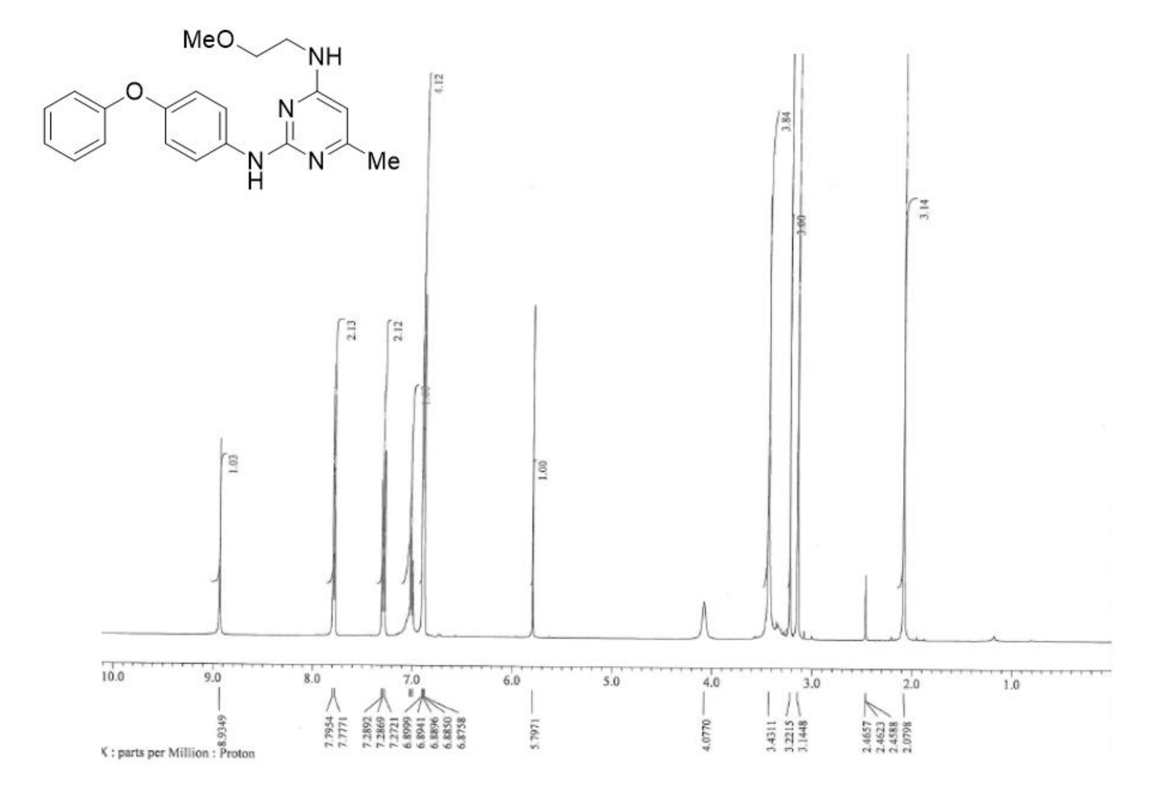

^{13}C-NMR (DMSO-d$_6$, 125 MHz)

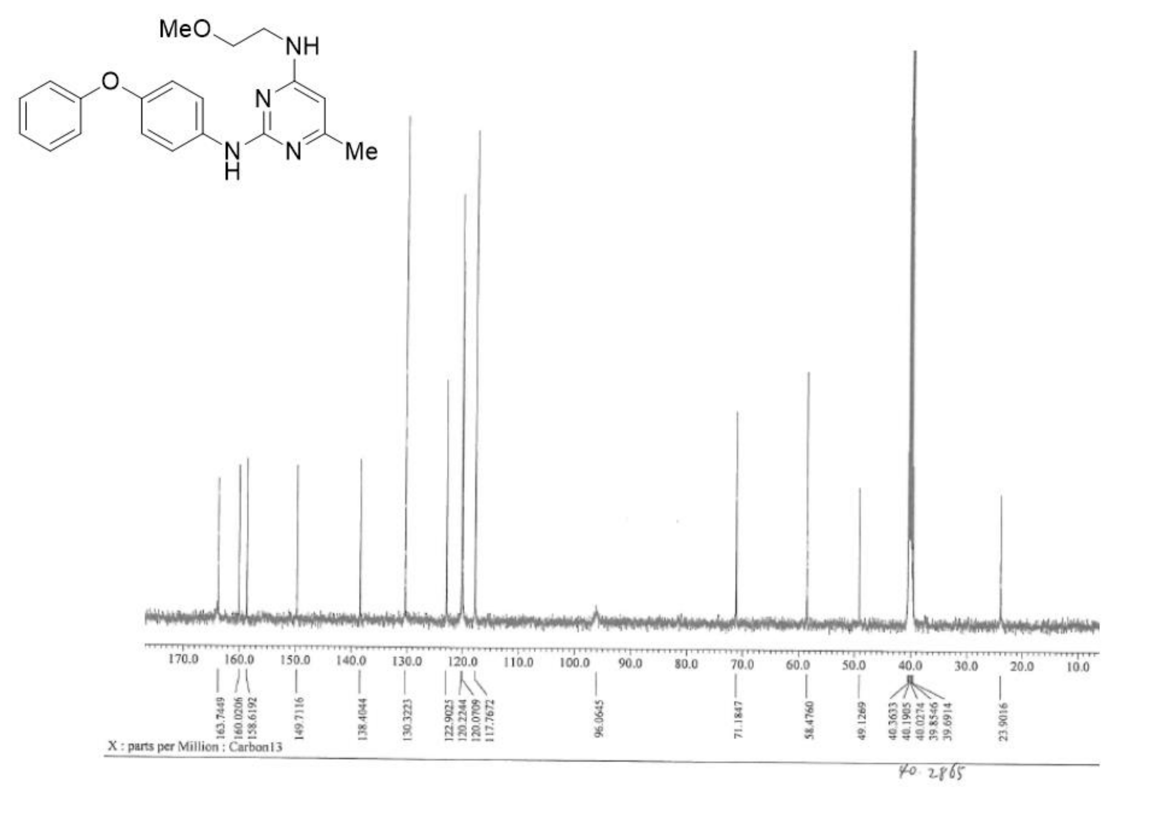

N[2]-(4-(4-chlorophenoxy)phenyl)-*N*[4]-(2-methoxyethyl)-6-methylpyrimidine-2,4-diamine (**Aa5**)

^{1}H-NMR (DMSO-d_6, 500 MHz)

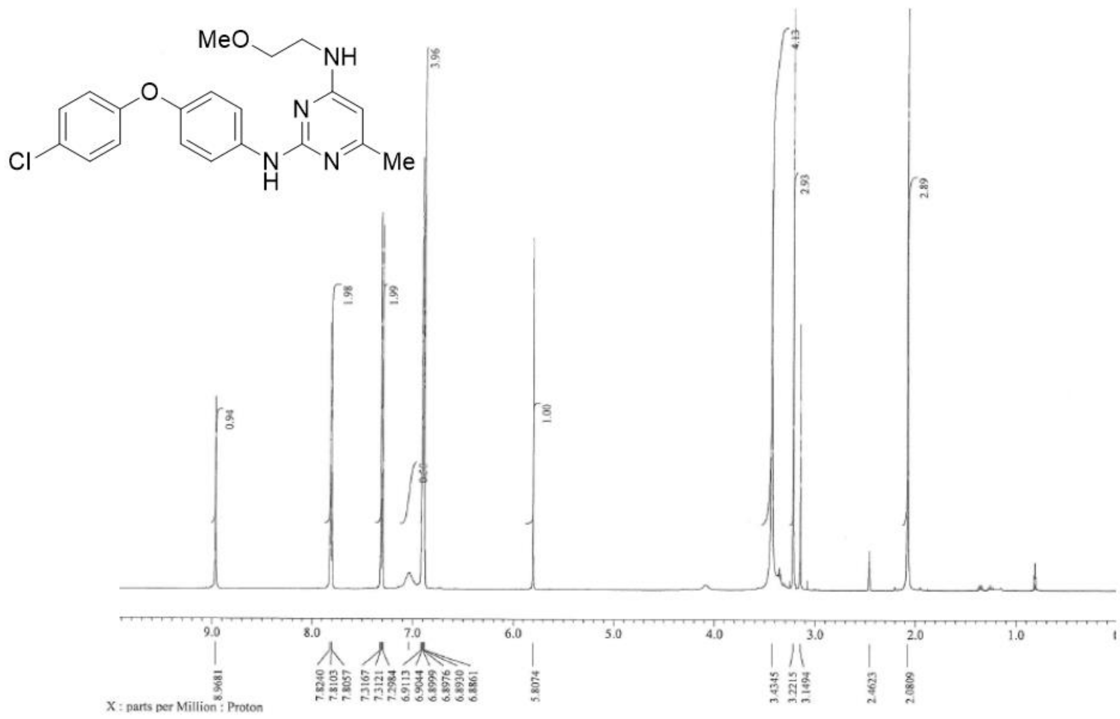

^{13}C-NMR (DMSO-d_6, 125 MHz)

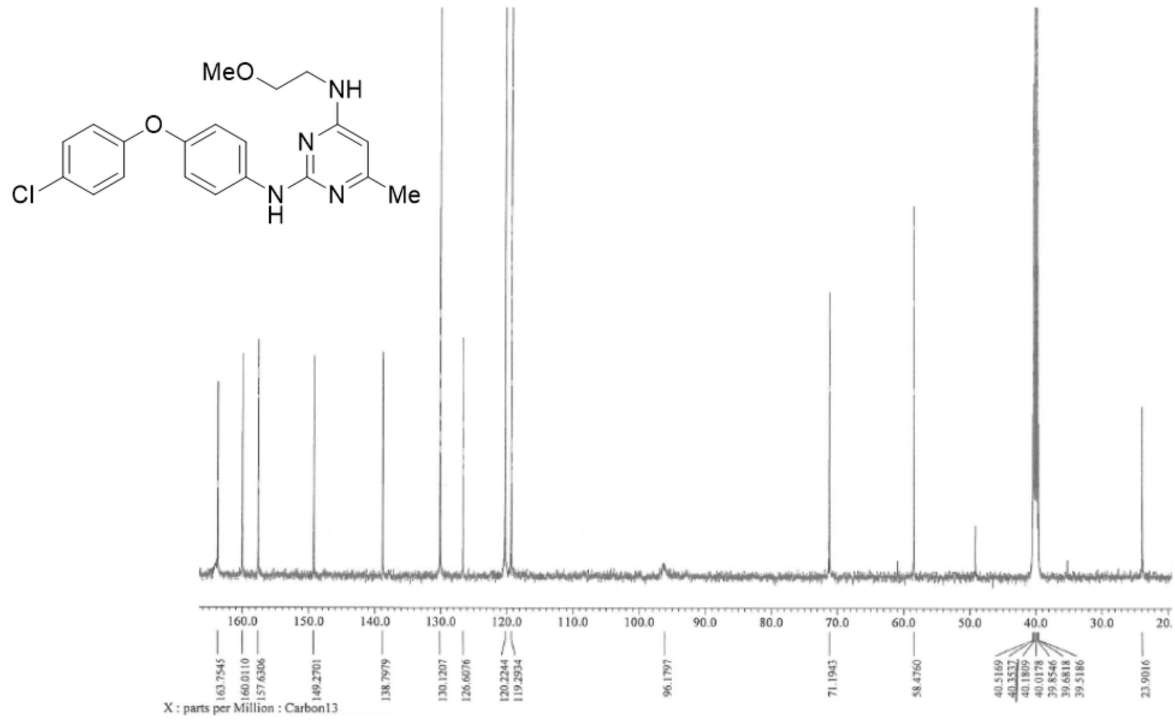

N[4]-(2-methoxyethyl)-6-methyl-*N*[2]-(4-(p-tolyloxy)phenyl)pyrimidine-2,4-diamine (**Aa6**)

^{1}H-NMR (DMSO-d$_6$, 500 MHz)

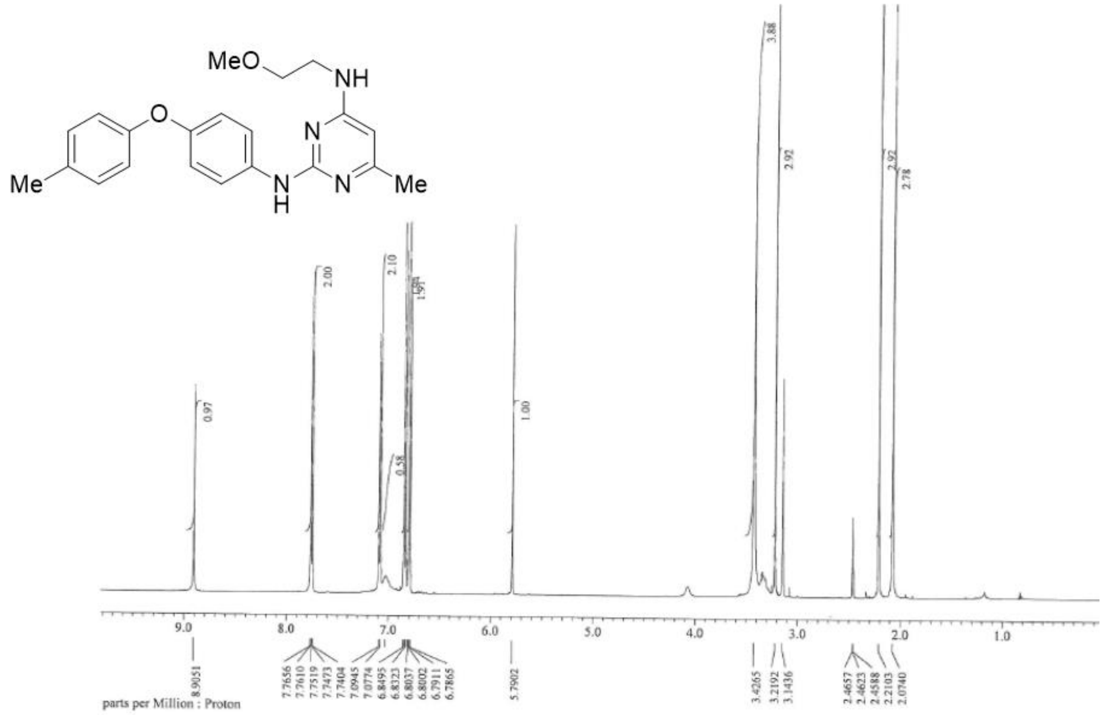

^{13}C-NMR (DMSO-d$_6$, 125 MHz)

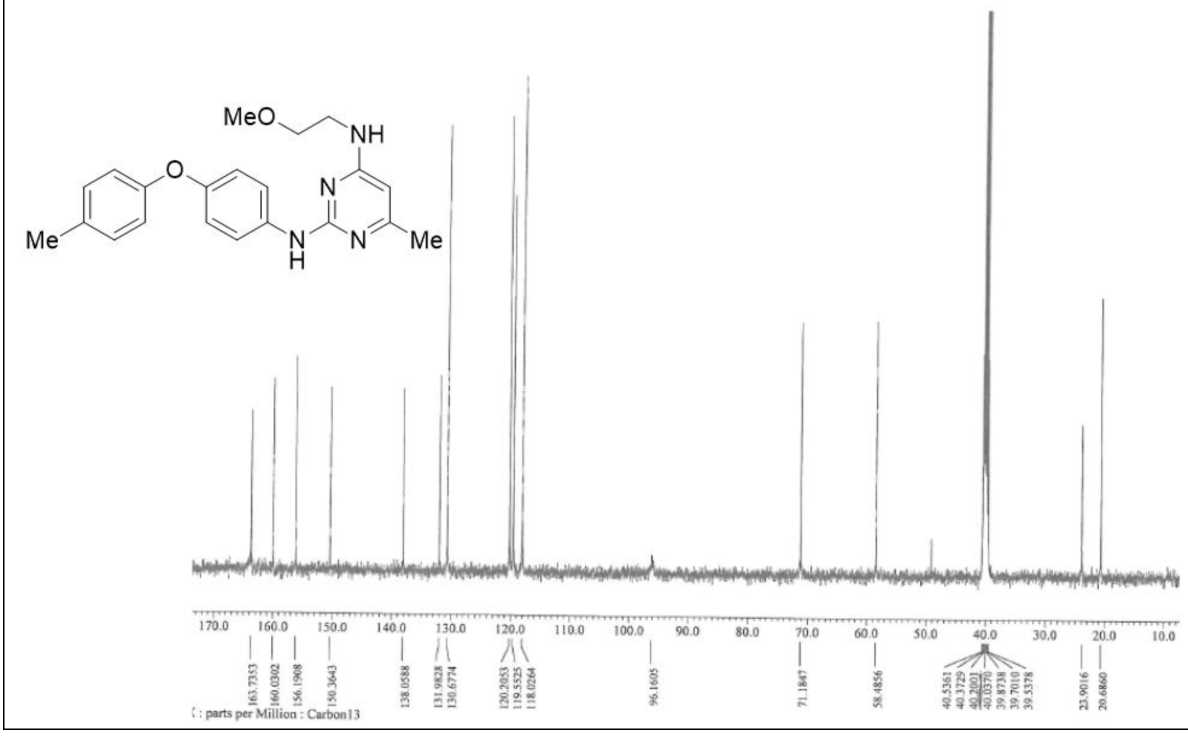

*N*4-(4-isopropoxyphenyl)-*N*2-(2-methoxyethyl)-6-methylpyrimidine-2,4-diamine (**Ba1**)

^{1}H-NMR (DMSO-d_6, 500 MHz)
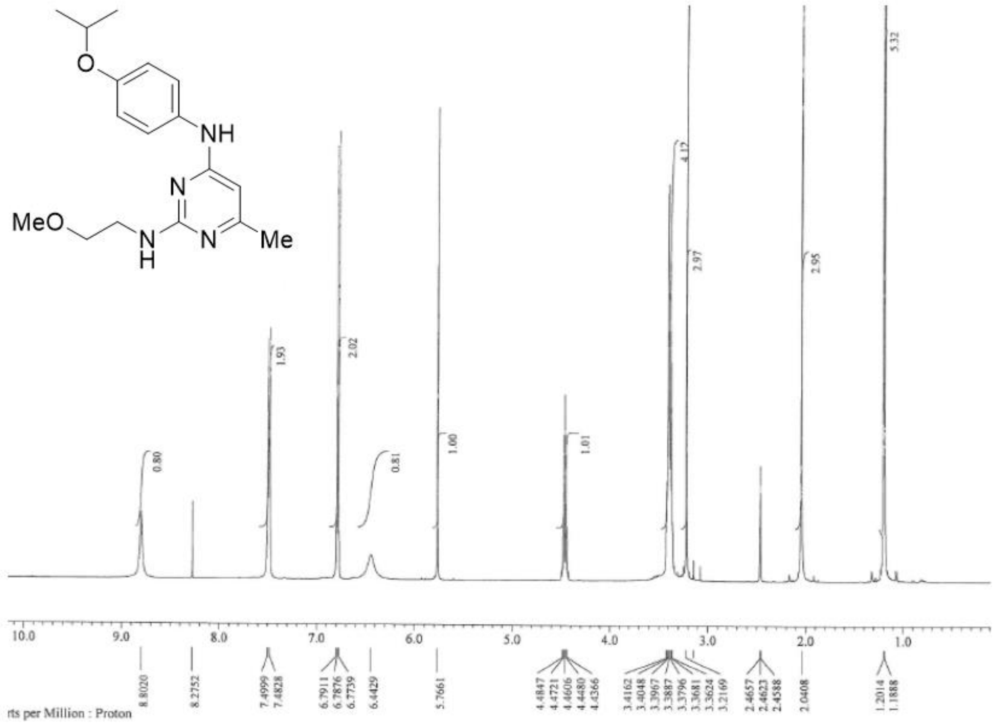
^{13}C-NMR (DMSO-d_6, 125 MHz)

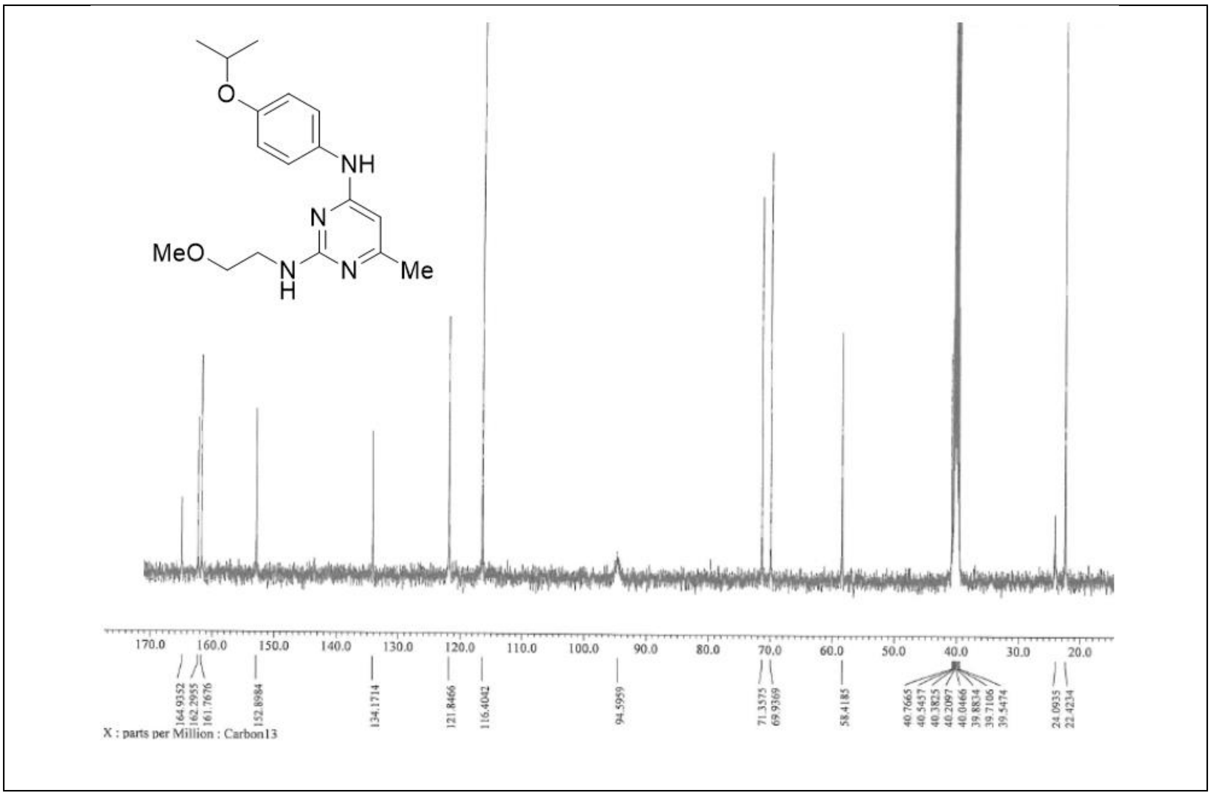

N^2-(2-methoxyethyl)-6-methyl-N^4-(4-morpholinophenyl)pyrimidine-2,4-diamine (**Ba2**).

^{1}H-NMR (DMSO-d$_6$, 500 MHz)

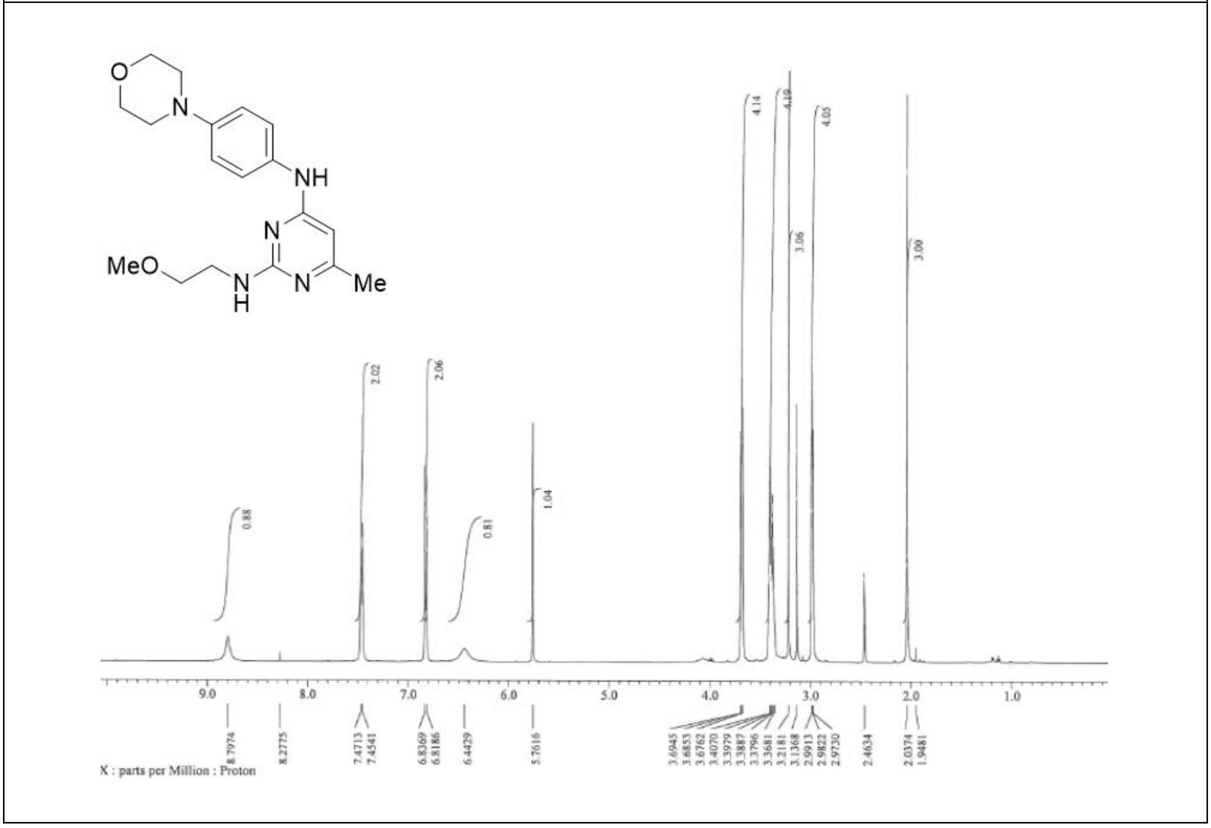

^{13}C-NMR (DMSO-d$_6$, 125 MHz)

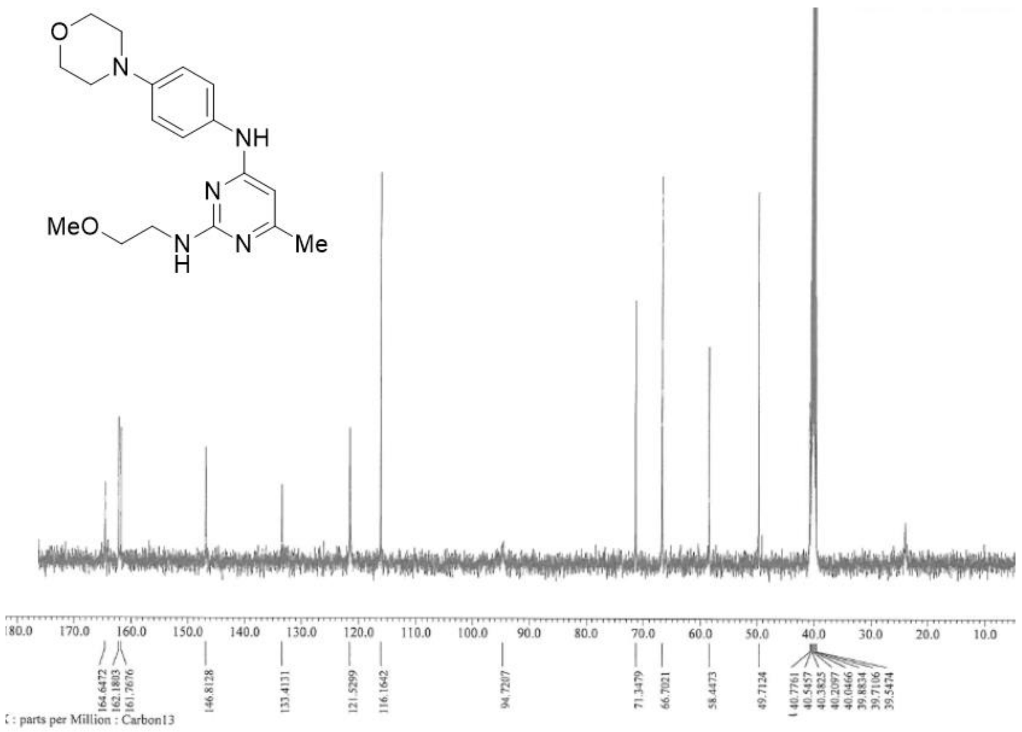

N[2]-(2-methoxyethyl)-6-methyl-*N*[4]-(4-(phenylamino)phenyl)pyrimidine-2,4-diamine (**Ba3**)

^{1}H-NMR (DMSO-d$_6$, 500 MHz)

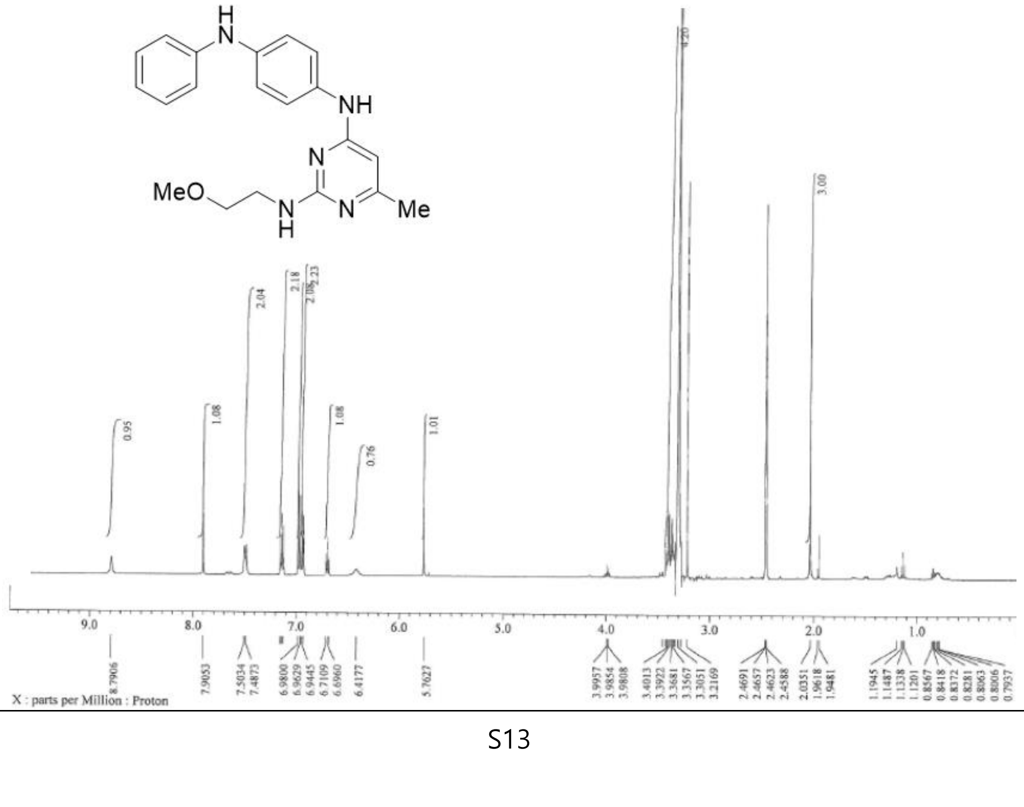

^{13}C-NMR (DMSO-d$_6$, 125 MHz)

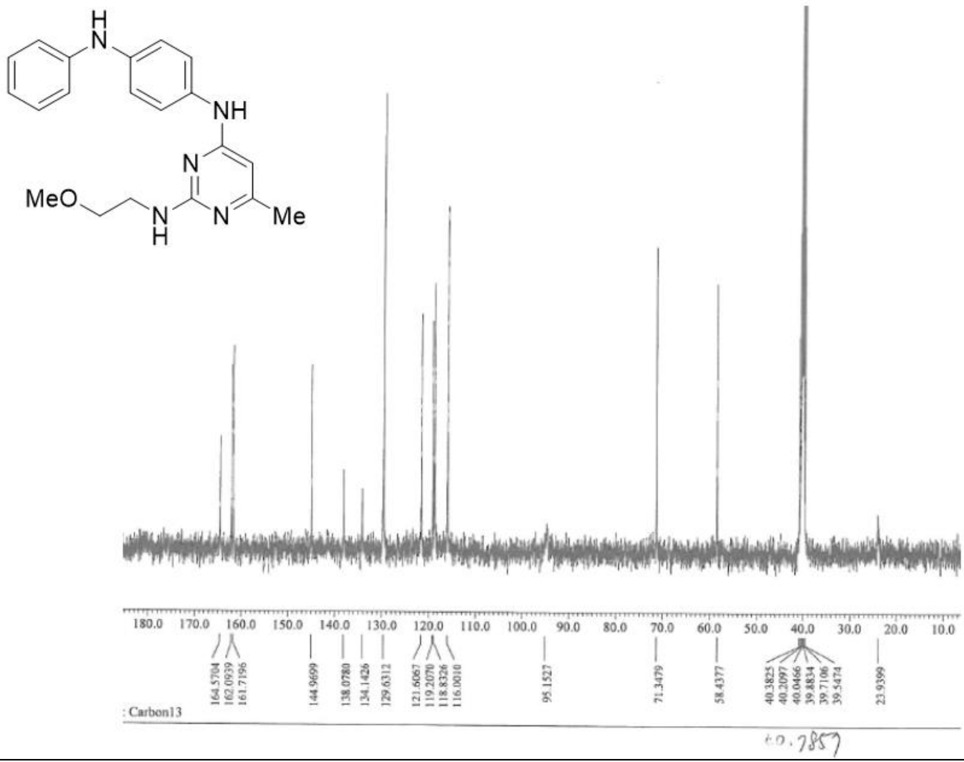

N^2-(2-methoxyethyl)-6-methyl-*N*^{4}-(4-phenoxyphenyl)pyrimidine-2,4-diamine (**Ba4**)

^{1}H-NMR (DMSO-d$_6$, 500 MHz)

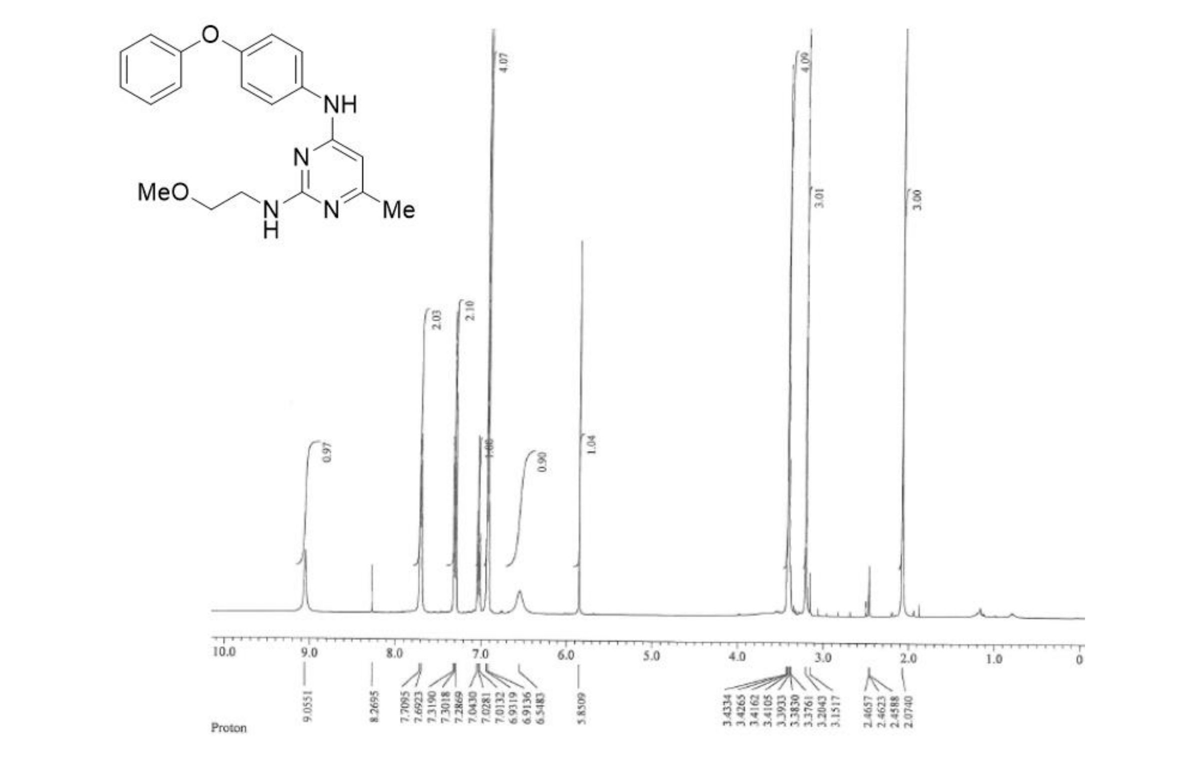

^{13}C-NMR (DMSO-d$_6$, 125 MHz)

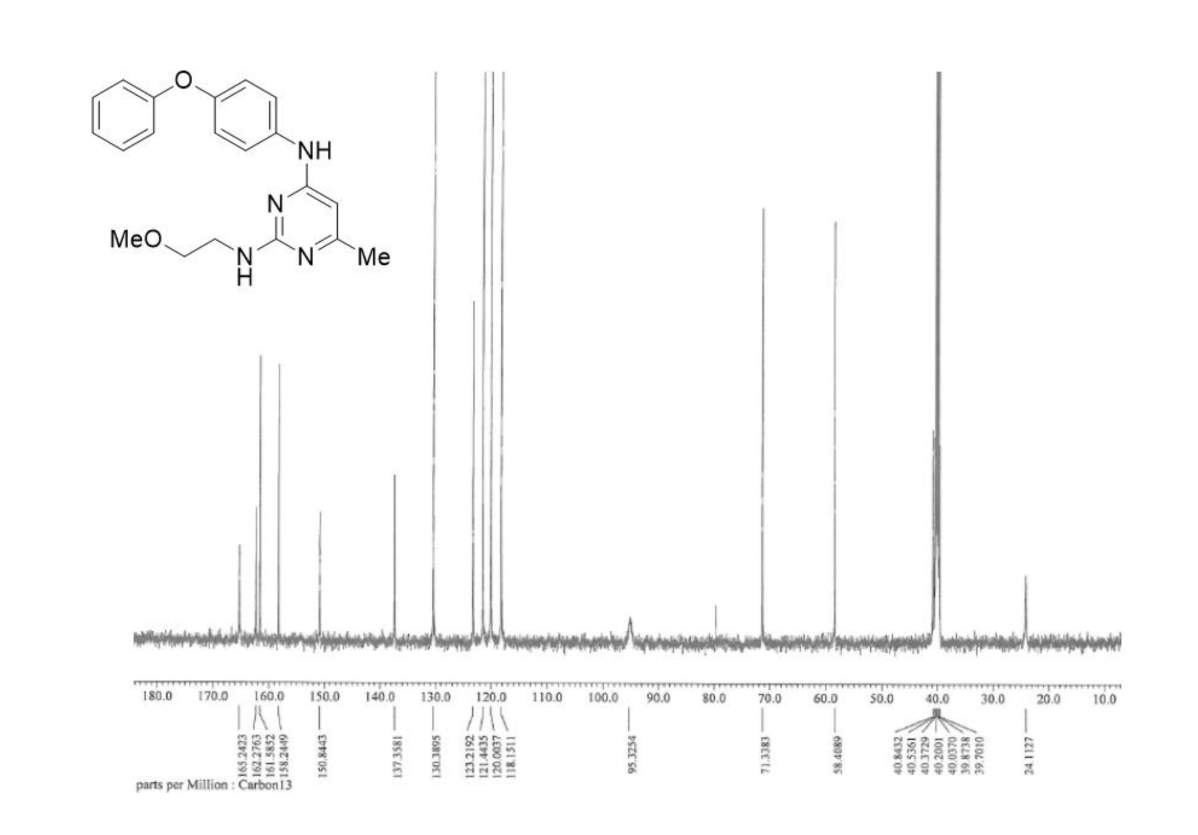

N[4]-(4-(4-chlorophenoxy)phenyl)-*N*[2]-(2-methoxyethyl)-6-methylpyrimidine-2,4-diamine (**Ba5**)

^{1}H-NMR (DMSO-d$_6$, 500 MHz)

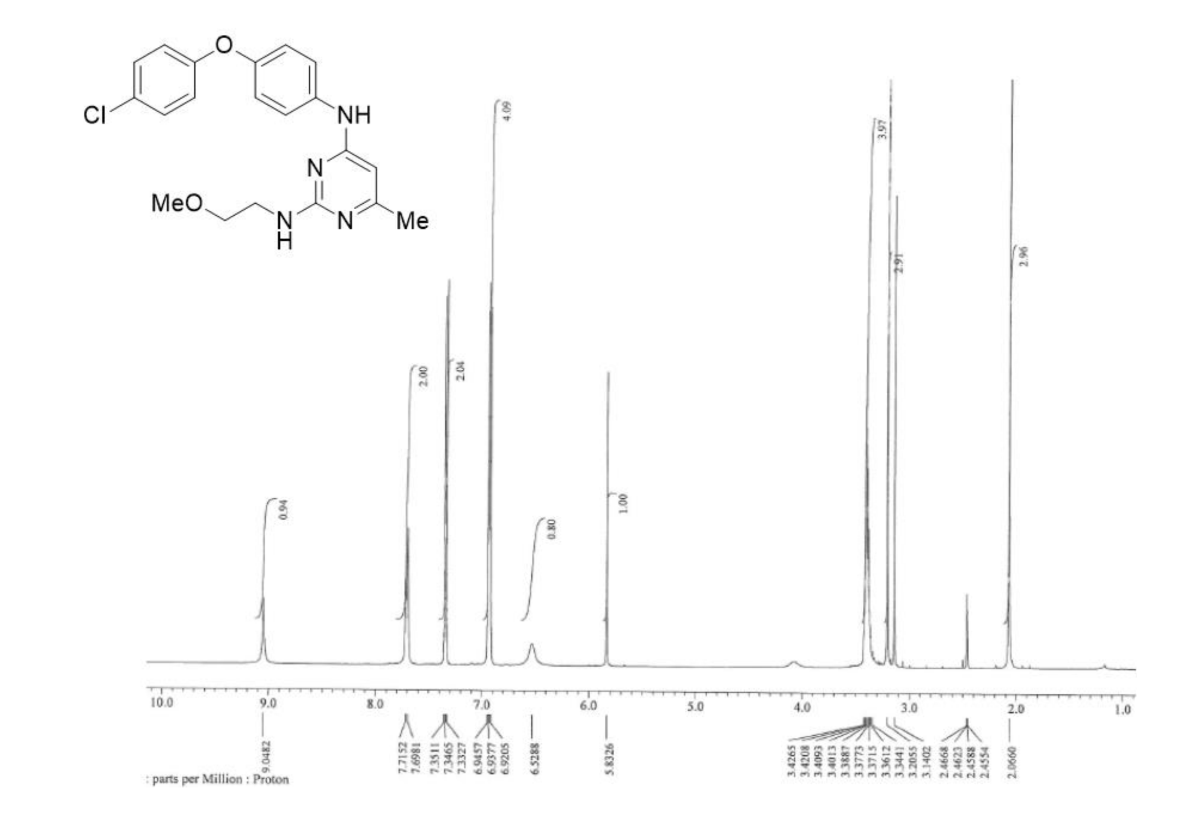

^{13}C-NMR (DMSO-d$_6$, 125 MHz)

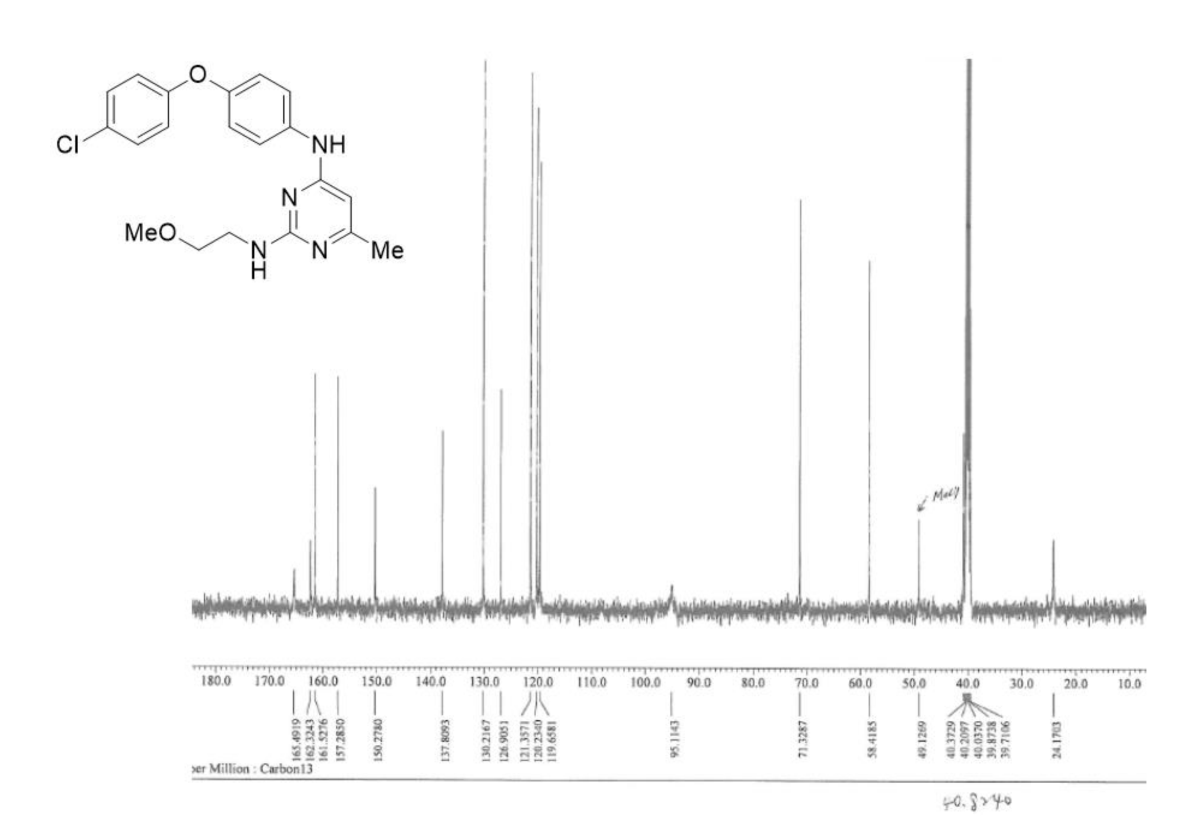

*N*2-(2-methoxyethyl)-6-methyl-*N*4-(4-(p-tolyloxy)phenyl)pyrimidine-2,4-diamine (**Ba6**)

^{1}H-NMR (DMSO-d$_6$, 500 MHz)

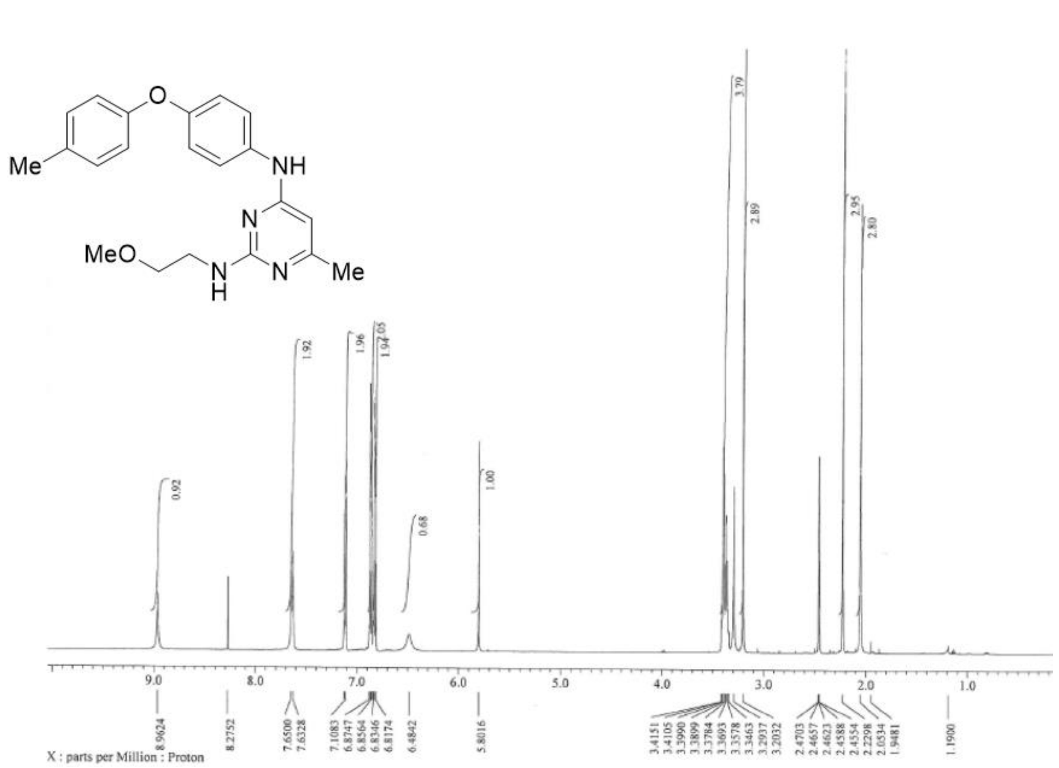

^{13}C-NMR (DMSO-d$_6$, 125 MHz)

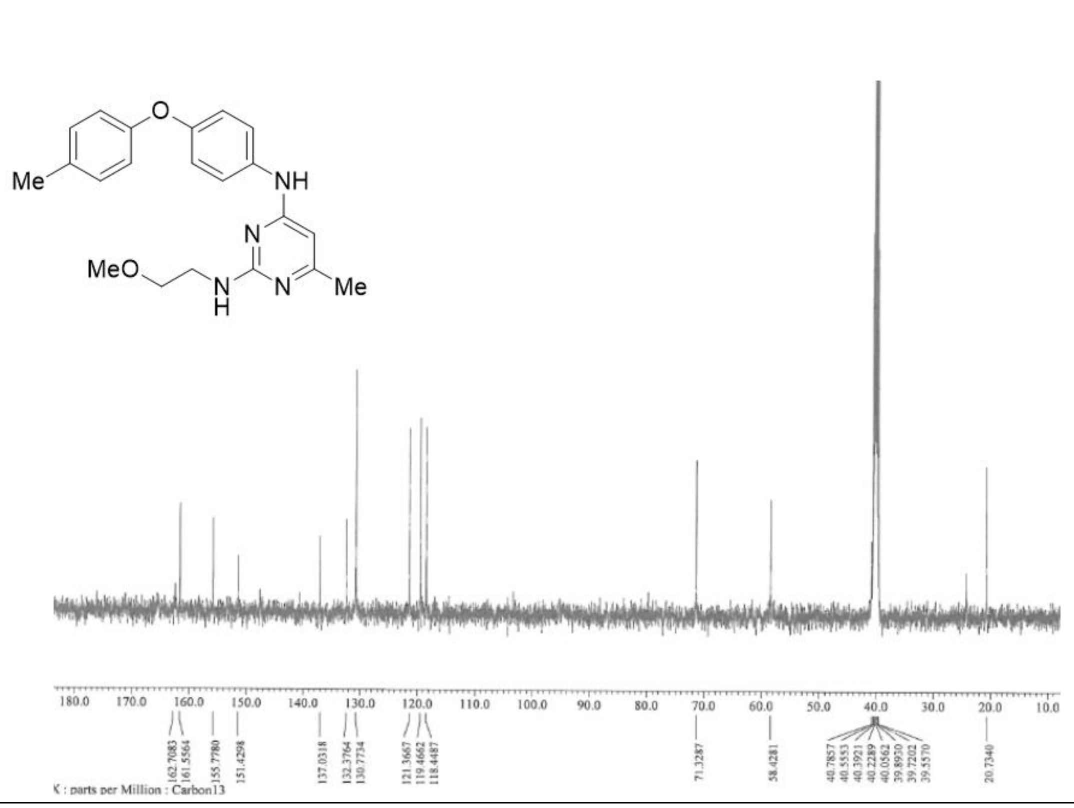

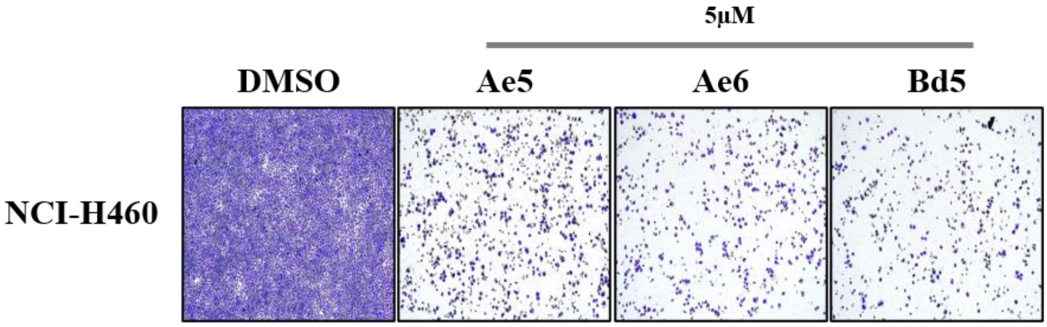

Figure 16. Cytotoxic activity for Bd5, Ae5 and Ae6 in NCI-H460 cells.

NCI-H460 was treated with **Bd5**, **Ae5** and **Ae6** at a concentration of 5 μM each, indicating the effect of ANO1 inhibition. After treatment for 24 h with each compound, the cells were fixed and stained using crystal violet for cell viability analysis. The result shows that **Bd5**, **Ae5** and **Ae6** were exhibited relatively high cytotoxicity compared with **Ae5**-treated cells, related to Figure 5a.

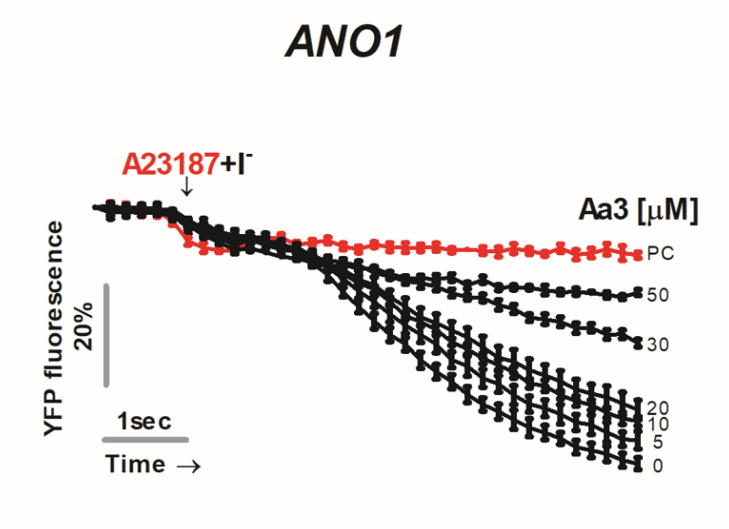

Figure S17. The Aa3 compound inhibits the A23187-induced ANO1 activity.

The biological activity of ANO1 was assessed in FRT-YFP-ANO1 cells. The cells were pre-treated with **Aa3** in a dose-dependent manner for 20 min, and then the ANO1 was activated with 20μM A23187, Ca^{2+} ionophore. PC; positive control (NPPB, known as chloride channel blocker). The result shows that the **Aa3** can block the biological activity of ANO1 without ATP stimulation increasing intracellular Ca^{2+} through P2Y-receptor pathway.

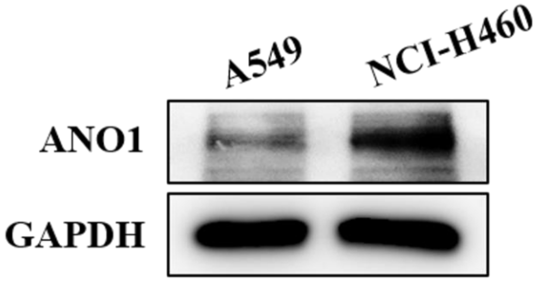

Figure 18. The expression levels of endogenous ANO1 protein between A549 and NCI-H460 cells.

The expression levels on endogenous ANO1 protein was evaluated by Western blotting. The result shows the different expression levels for endogenous ANO1. NCI-H460 cells highly express the ANO1, whereas A549 cells show relatively lower expression levels of ANO1.
